# Oncoprotein CYB561, acting in IRE1-XBP1-SREBF1 and FAK-ERK pathway, promotes breast cancer lipogenesis and progression

**DOI:** 10.1038/s41420-026-03101-2

**Published:** 2026-04-13

**Authors:** Xiaochen Yang, Yukai Tao, Yan Xu, Qixiang Shao, Guoqin Jiang

**Affiliations:** 1 Department of Thyroid and Breast Surgery, Affiliated Kunshan Hospital of Jiangsu University, Kunshan, PR China; 2 Clinical Research & Lab Center, Affiliated Kunshan Hospital of Jiangsu University, Kunshan, PR China; 3 Department of Immunology, Institute of Medical Genetics and Reproductive Immunity, Jiangsu Province Engineering Research Center of Precise Prevention in the Digestive and Reproductive System Cancers, School of Medical Science and Laboratory Medicine, Jiangsu College of Nursing, Huai’an, PR China; 4 Department of Surgery, The Second Affiliated Hospital of Soochow University, Suzhou, PR China

**Keywords:** Breast cancer, Breast cancer

## Abstract

Breast cancer (BC) heterogeneity, particularly in triple-negative (TNBC) and HER2-positive subtypes, underpins therapeutic challenges and cancer-related mortality worldwide. Here, we identify the transmembrane oxidoreductase CYB561 as a pivotal oncoprotein that drives BC malignancy by coordinating lipid metabolic reprogramming and oncogenic signaling. CYB561 was significantly overexpressed in BC tissues, especially in HER2-positive (70%) and TNBC (60%) cases, and its expression correlated with advanced T stage, metastasis, and poor patient survival. Functionally, CYB561 potently promoted proliferation, migration, invasion, epithelial-mesenchymal transition (EMT), and tumor growth in vitro and in vivo. Mechanistically, CYB561 directly bound IRE1, triggering the IRE1–XBP1–SREBF1 axis to induce lipogenic enzyme expression and lipid droplet accumulation. Concurrently, it activated the FAK–ERK signaling pathway. Crucially, IRE1 knockdown abolished CYB561-induced lipogenesis and malignant phenotypes, while ERK inhibition partially attenuated these effects, revealing a synergistic crosstalk between metabolic and signaling axes. Our findings establish CYB561 as a master regulator of BC progression and nominate it as a promising therapeutic target for aggressive breast cancers.

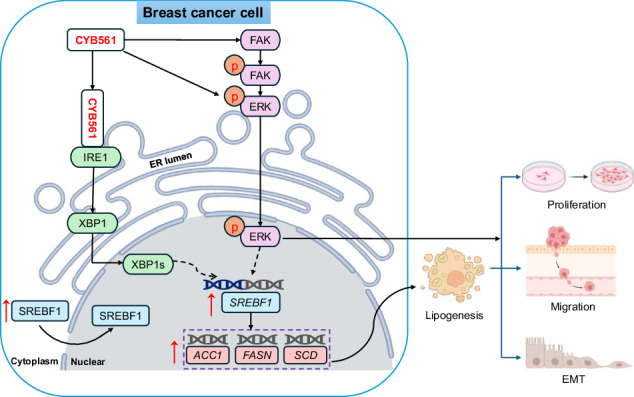

## Introduction

Breast cancer (BC) persists as the most prevalent malignancy among women worldwide [1], with triple-negative (TNBC) and HER2-positive subtypes presenting formidable therapeutic challenges due to their aggressive clinical course and limited treatment options [2, 3]. While targeted therapies like trastuzumab have improved outcomes, intrinsic and acquired resistance remain significant barriers, underscoring the urgent need to identify novel drivers of progression and resistance [4].

A key vulnerability of many aggressive tumors, including BC, lies in their rewired metabolic programs. The dysregulation of lipid metabolism, in particular, has emerged as a critical facilitator of tumor growth, metastasis, and therapy resistance [5]. Cancer cells enhance de novo lipogenesis to supply essential building blocks for membranes and signaling molecules, with key enzymes like FASN and ACC1 becoming crucial for their survival and proliferation [6, 7]. This metabolic dependency holds promise as a breakthrough therapeutic approach for cancer [7–9], yet the upstream molecular triggers that initiate and sustain this rewiring in BC are not fully understood.

The transmembrane oxidoreductase CYB561 has recently gained attention in oncology. Recent work from our group and others reveals that CYB561 is overexpressed in BC tissues, particularly in aggressive subtypes, and its high expression correlates with advanced disease and poor survival [10, 11]. Studies also have begun to delineate its oncogenic roles, linking it to the potentiation of Akt/mTOR signaling in HER2-positive models and the modulation of NF-κB pathways [12, 13]. However, a significant gap remains as whether and how CYB561 contributes to the metabolic alterations, particularly the lipid and energy reprogramming, that fuel BC malignancy is still unclear.

Herein, we set out to determine whether CYB561 acts as a metabolic master regulator in BC. Our investigation reveals that CYB561 is a central node connecting two powerful oncogenic circuits: the IRE1-XBP1-SREBF1 lipogenic axis and the FAK-ERK signaling pathway. We demonstrate that CYB561-driven metabolic reprogramming is a fundamental mechanism underlying its promotion of proliferation, migration, EMT, and tumor growth across molecular subtypes. These findings not only unveil a novel function for CYB561 but also identify it as a compelling therapeutic target for aggressive breast cancers.

## Results

### Elevated expression of CYB561 in BC

**Fig. 1:**
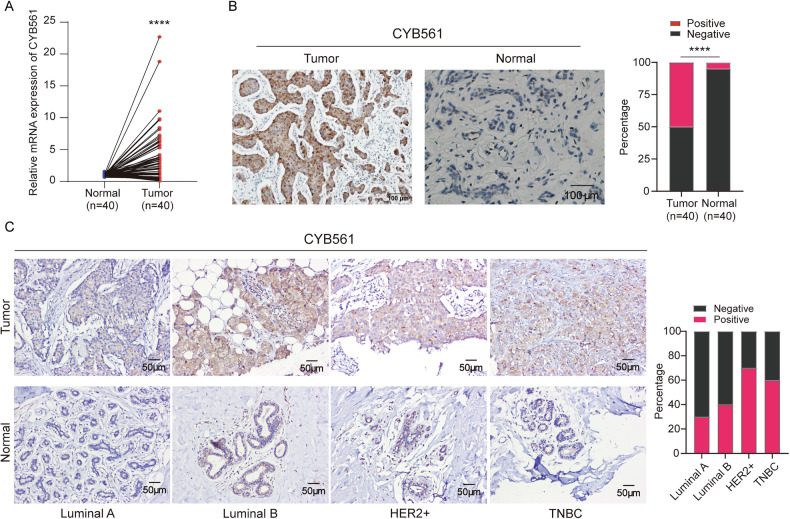
Elevated expression of CYB561 in breast cancer tissues. **A** RT-qPCR analysis of 40 paired fresh breast cancer and para-tumor normal tissues revealed significantly higher CYB561 mRNA expression in tumor tissues. **B** Immunohistochemistry showed a markedly higher positive rate of CYB561 protein in cancer tissues (50%, 20/40) compared to para-tumor normal tissues (5%, 2/40). **C** CYB561 protein positivity rates by molecular subtype: HER2-positive (70%), TNBC (60%), Luminal A (30%), and Luminal B (40%). Data were presented as mean ± standard deviation (SD, *n* = 3). **** *P* < 0.0001.

Building on previous TCGA and GEO analyses linking high CYB561 expression to poor prognosis in BC [10], we further evaluated CYB561 levels in 40 paired tumor and para-tumor normal tissues from surgical patients. RT-qPCR confirmed significantly higher CYB561 mRNA levels in tumor tissues compared to normal tissues adjacent to tumor (*P* < 0.0001, Fig. 1A). Immunohistochemistry showed CYB561 protein positivity in 50% of tumor specimens versus only 5% of para-tumor normal tissues (Fig. 1B), with cytoplasmic localization. Subtype analysis revealed particularly high positivity in HER2-positive (70%) and TNBC (60%) subtypes (Fig. 1C), corroborating earlier reports [12, 13] and underscoring the association between CYB561 overexpression and aggressive BC subtypes.

### CYB561 regulates BC cell proliferation and tumor growth in vitro and in vivo

**Fig. 2:**
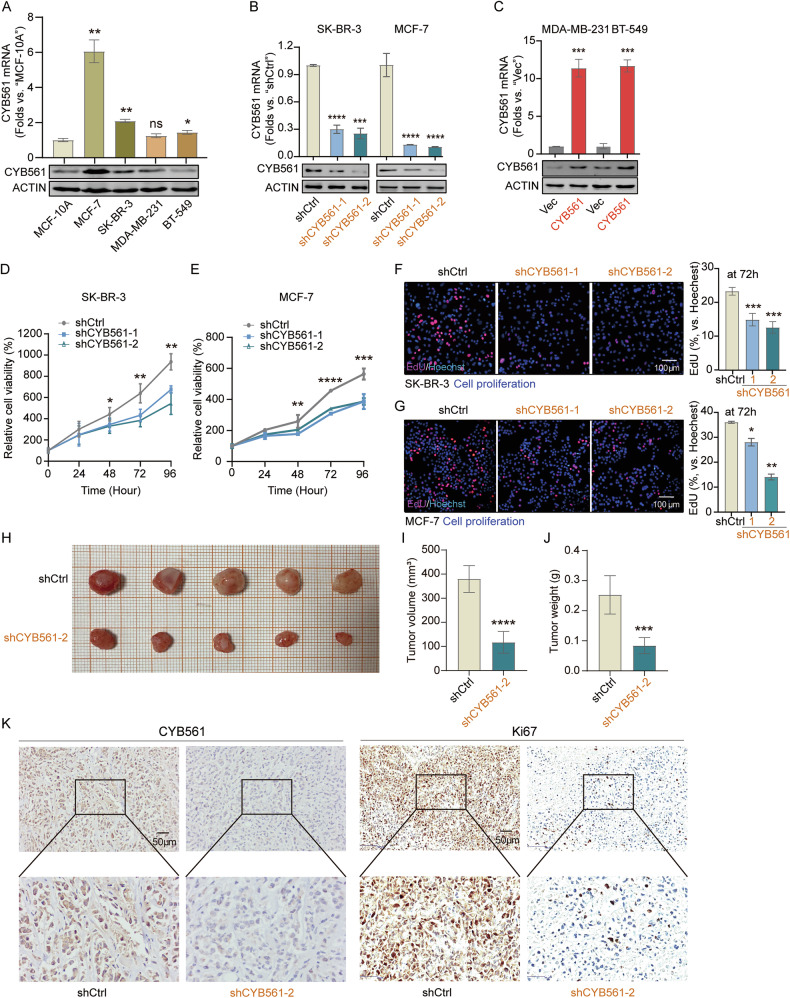
Knockdown of CYB561 inhibits breast cancer cell proliferation and tumor growth in vitro and in vivo. **A** Expression levels of CYB561 in normal mammary epithelial cells and breast cancer cell lines. **B** Stable CYB561 knockdown models were established in SK-BR-3 and MCF-7 cells using lentiviral vectors expressing two distinct shRNAs (shCYB561-1 and shCYB561-2), with a non-targeting scramble RNA (shCtrl) as control. Knockdown efficiency was confirmed by qPCR and Western blot (WB). **C** Stable CYB561 overexpression models were generated in MDA-MB-231 and BT-549 cells *via* lentiviral transduction (CYB561 group), with empty vector (Vec) as control. Overexpression efficiency was verified by qPCR and WB. After culturing for the designated hours, CCK-8 assays (**D**, **E**) and EdU assays (**F**, **G**) were performed. SK-BR-3 cells stably expressing either control shRNA (shCtrl) or shCYB561-2 were subcutaneously injected into 4–5-week-old female BALB/c nude mice to establish xenograft models. Mice were euthanized after 40 days, and tumors were excised. Representative tumor images (**H**), final tumor volumes (**I**), and tumor weights (**J**) are shown, with comparisons between groups. **K** Representative immunohistochemical staining of CYB561 and Ki67 in tumor tissues from the shCtrl and shCYB561-2 groups. Data were presented as mean ± standard deviation (SD). * *P* < 0.05, ** *P* < 0.01, *** *P* < 0.001, **** *P* < 0.0001. “ns” stands for *P* > 0.05. In vitro experiments in this figure were repeated three times.

**Fig. 3:**
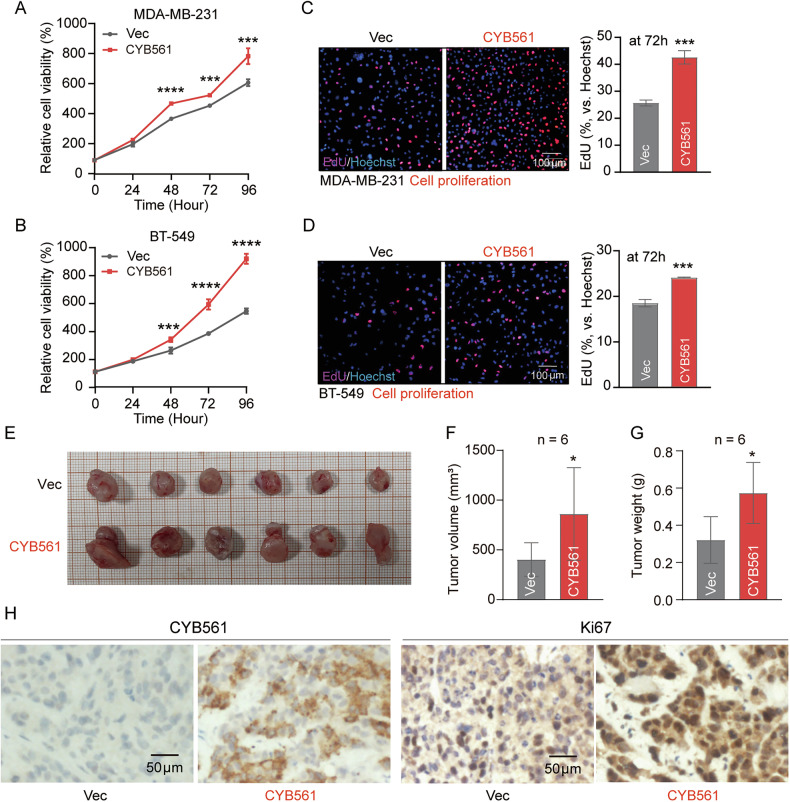
CYB561 promotes breast cancer cell proliferation and tumor growth in vitro and in vivo. **A**, **B** CCK-8 assays and **C**, **D** EdU assays were performed after the indicated culture time in MDA-MB-231 and BT-549 cells stably overexpressing CYB561 (CYB561 group) or empty vector (Vec group) established *via* lentiviral transduction. MDA-MB-231 cells stably expressing empty vector (Vec) or CYB561 were subcutaneously injected into 4–5-week-old female BALB/c nude mice to establish xenograft tumor models. The mice were euthanized after 35 days, and tumors were harvested. Representative tumor images (**E**), end-point tumor volume (**F**), and end-point tumor weight (**G**) are shown, with comparisons between groups. **H** Representative immunohistochemical staining of CYB561 and Ki67 in tumor tissues from the Vec and CYB561 groups. Data were presented as mean ± standard deviation (SD). * *P* < 0.05, *** *P* < 0.001, **** *P* < 0.0001. In vitro experiments in this figure were repeated three times.

To validate above results solidly, we first assessed basal CYB561 expression across breast cell lines, noting high levels in SK-BR-3 and MCF-7 cells and relatively low expression in MDA-MB-231 and BT-549 cells compared to non-tumorigenic MCF-10 A cells (Fig. 2A). To functionally assess the role of CYB561, loss-of-function studies using shRNA-mediated knockdown in SK-BR-3 and MCF-7 cells (Fig. 2B) and gain-of-function studies *via* overexpression in MDA-MB-231 and BT-549 cells (Fig. 2C) were conducted. Knockdown of CYB561 markedly suppressed cell proliferation in vitro (Fig. 2D–G) and delayed tumor growth in xenograft models (Fig. 2H–J), accompanied by reduced Ki-67 expression (Fig. 2K). Conversely, CYB561 overexpression enhanced proliferation in vitro (Fig. 3A–D) and promoted larger tumor formation in vivo, with increased Ki-67 signal (Fig. 3E–H).

### CYB561 induces EMT and enhances cell motility

**Fig. 4:**
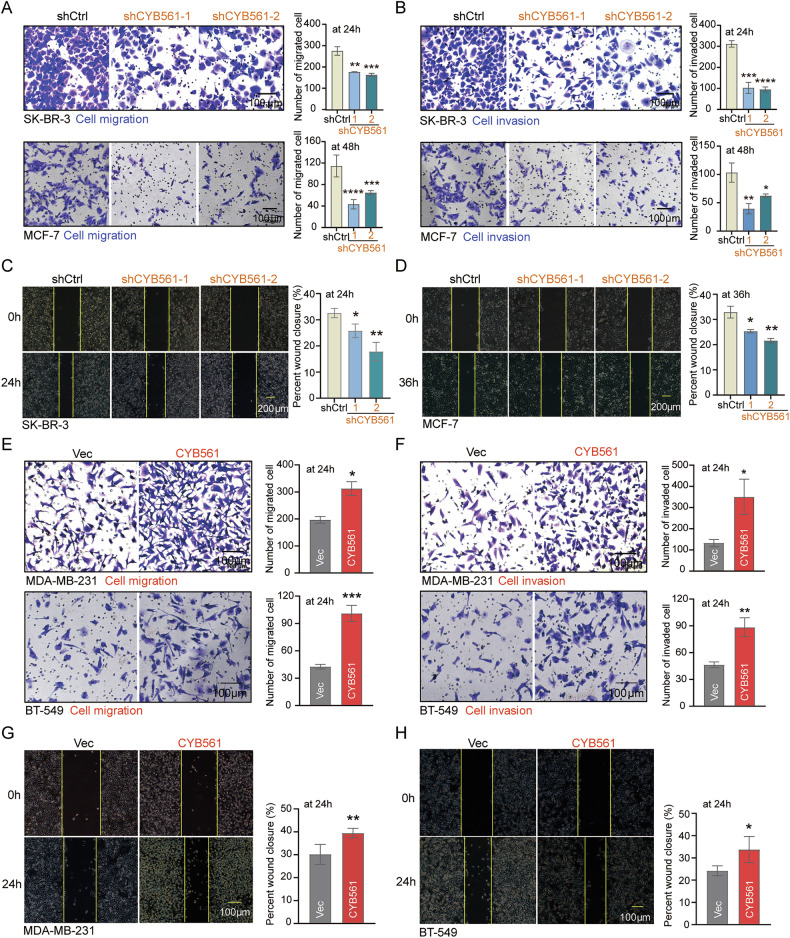
CYB561 promotes breast cancer cell migration and invasion. **A**, **B** Transwell and **C**, **D** wound healing assays were performed in SK-BR-3 and MCF-7 cells with stable CYB561 knockdown (using two distinct targeting sequences: shCYB561-1 and shCYB561-2) or scramble RNA control (shCtrl) introduced *via* lentiviral vectors. **E**, **F** Transwell and **G**, **H** wound healing assays were conducted in MDA-MB-231 and BT-549 cells stably overexpressing CYB561 (CYB561 group) or empty vector (Vec group) established using lentiviral vectors. Data were presented as mean ± standard deviation (SD, *n* = 3). * *P* < 0.05, ** *P* < 0.01, *** *P* < 0.001, **** *P* < 0.0001. Experiments in this figure were repeated three times.

To explore the biological processes associated with CYB561, we performed Gene Ontology (GO) analysis of CYB561-associated differentially expressed genes, which revealed significant enrichment in cell–cell adhesion and extracellular matrix organization pathways (Supplementary Fig. 1). This prompted us to investigate whether CYB561 regulates EMT. shRNA-mediated knockdown of CYB561 in SK-BR-3 and MCF-7 cells markedly impaired migratory and invasive capacities in both Transwell and wound healing assays (Fig. 4A–D). Conversely, in gain-of-function models, stable overexpression of CYB561 in MDA-MB-231 and BT-549 cells significantly enhanced these abilities (Fig. 4E–H).

**Fig. 5:**
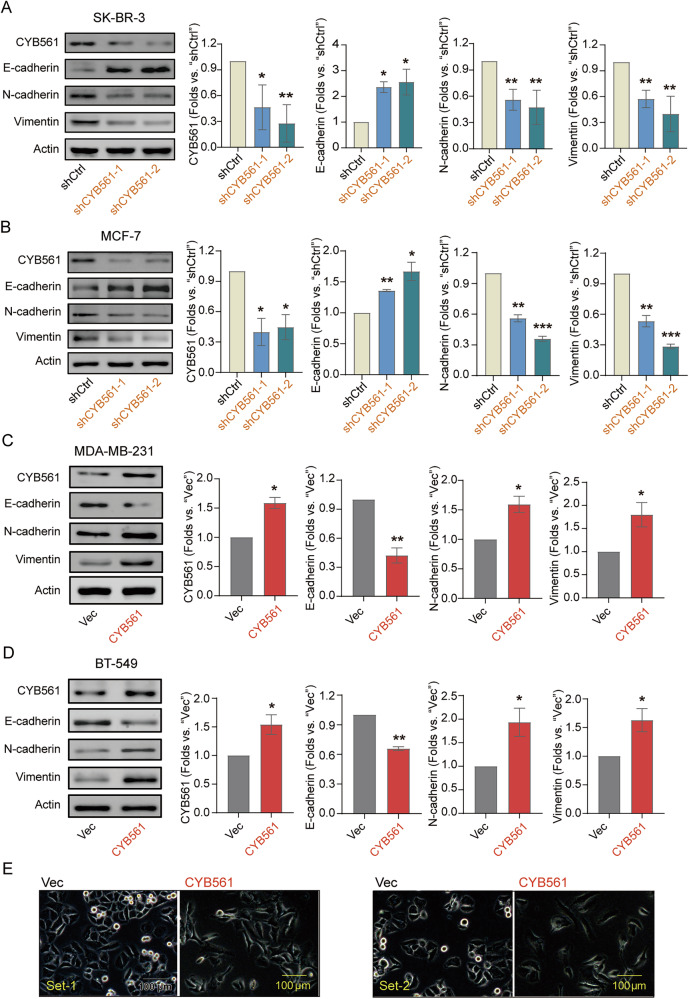
CYB561 promotes breast cancer cell EMT. **A**, **B** Western blot analysis of key epithelial-mesenchymal transition (EMT) proteins in SK-BR-3 (**A**) and MCF-7 (**B**) cells with stable CYB561 knockdown (using two distinct shRNAs: shCYB561-1 and shCYB561-2) or scramble RNA control (shCtrl) introduced *via* lentiviral vectors. **C**, **D** Western blot analysis of key EMT proteins in MDA-MB-231 (**C**) and BT-549 (**D**) cells stably overexpressing CYB561 (CYB561 group) or empty vector (Vec group) established *via* lentiviral transduction. **E** Representative morphological changes associated with EMT in CYB561-overexpressed SK-BR-3 breast cancer cells. Data were presented as mean ± standard deviation (SD, *n* = 3). * *P* < 0.05, ** *P* < 0.01, *** *P* < 0.001. Experiments in this figure were repeated three times. Scale bar =100 μm.

At the molecular level, knockdown of CYB561 in SK-BR-3 and MCF-7 cells upregulated the epithelial marker E-cadherin and downregulated mesenchymal markers N-cadherin and Vimentin (Fig. 5A, B). Conversely, CYB561 overexpression in MDA-MB-231 and BT-549 cells produced the opposite effect, with decreased E-cadherin and increased N-cadherin and Vimentin expression (Fig. 5C, D). Morphological examination further revealed that CYB561-overexpressing SK-BR-3 cells adopted a spindle-shaped, fibroblast-like appearance characteristic of mesenchymal cells (Fig. 5E). Collectively, these findings demonstrate that CYB561 promotes EMT and enhances cell motility in a context-independent manner across multiple BC subtypes.

### CYB561 drives de novo lipogenesis and lipid synthesis is causally required for CYB561-induced EMT

**Fig. 6:**
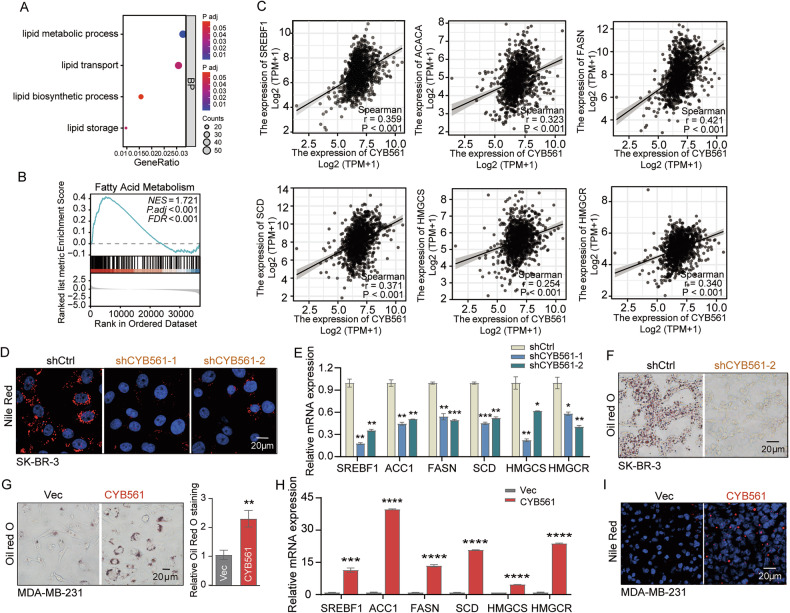
CYB561 drives de novo lipogenesis in breast cancer cells. **A** GO and **B** GSEA analyses of differentially expressed genes between high and low CYB561 expression groups in the TCGA-BRCA cohort. **C** Correlation analysis between CYB561 expression and SREBF1 or other key lipogenic enzyme genes in the TCGA-BRCA dataset. **D** Lipid droplet content was assessed by Nile Red staining in SK-BR-3 cells with stable CYB561 knockdown (using two distinct shRNAs: shCYB561-1 and shCYB561-2) or control (shCtrl), established *via* lentiviral vectors. **E** RT-qPCR analysis of mRNA expression levels of key lipogenic enzymes in SK-BR-3 cells with stable CYB561 knockdown (shCYB561-1/shCYB561-2) or control (shCtrl), established *via* lentiviral vectors. **F** Frozen sections of tumor tissues from subcutaneous xenograft models in nude mice were stained with Oil Red O to evaluate lipid droplet content in CYB561-knockdown tumors. **G** Lipid droplet content was detected by Oil Red O staining in MDA-MB-231 cells stably overexpressing CYB561 (CYB561 group) or empty vector (Vec group) *via* lentiviral transduction. **H** RT-qPCR analysis of mRNA expression levels of key lipogenic enzymes in MDA-MB-231 cells stably overexpressing CYB561 (CYB561 group) or empty vector (Vec group), established *via* lentiviral transduction. **I** Frozen sections of tumor tissues from subcutaneous xenograft models were subjected to Nile Red staining to analyze lipid droplet content in CYB561-overexpressing tumors. Data were presented as mean ± standard deviation (SD, *n* = 3). * *P* < 0.05, ** *P* < 0.01, *** *P* < 0.001, **** *P* < 0.0001. “BP” stands for biological process. Experiments in this figure were repeated three times.

Given prior evidence of CYB561’s involvement in lipid metabolism [10], we first performed GO and Gene Set Enrichment Analysis (GSEA) on TCGA-BRCA data. Tumors with high CYB561 expression showed significant enrichment in lipid biosynthetic and fatty acid metabolic pathways (Fig. 6A, B), and CYB561 levels positively correlated with multiple lipogenic genes, including *SREBF1*, *FASN*, *ACC1*, and *SCD* across the cohort (Fig. 6C). These bioinformatic findings prompted us to investigate whether CYB561 directly regulates de novo lipogenesis in BC cells.

Consistent with the computational predictions, shRNA-mediated knockdown of CYB561 in SK-BR-3 and MCF-7 cells markedly reduced intracellular neutral lipid accumulation, as evidenced by Nile Red and Oil Red O staining (Fig. 6D and Supplementary Fig. 2A). This reduction was accompanied by significant downregulation of key lipogenic enzymes, including *SREBF1*, *ACC1*, *FASN*, *SCD*, *HMGCS*, and *HMGCR* (Fig. 6E). Moreover, xenograft tumors derived from CYB561-knockdown cells exhibited minimal lipid droplet deposition in frozen sections (Fig. 6F and Supplementary Fig. 2B), confirming the in vivo relevance of this regulation.

Conversely, stable overexpression of CYB561 in MDA-MB-231 and BT-549 cells robustly enhanced lipid droplet formation (Fig. 6G and Supplementary Fig. 2C) and upregulated the same panel of lipogenic genes (Fig. 6H). Xenograft tumors derived from CYB561-overexpressing cells exhibited abundant lipid droplets upon Nile Red and Oil Red O staining of frozen sections (Fig. 6I and Supplementary Fig. 2D), further supporting a conserved role for CYB561 in promoting lipogenesis across diverse BC subtypes.

Notably, emerging evidence implicates de novo lipogenesis as a key enabler of EMT [14]. Given that CYB561 robustly drives both lipogenesis and EMT, we next tested whether its lipogenic activity is functionally required for the induction of mesenchymal traits. Pharmacological inhibition of FASN with C75 abrogated both lipid accumulation and the EMT program in CYB561-overexpressing cells (Supplementary Fig. 3A, B), while palmitic acid supplementation largely rescued the EMT markers despite FASN inhibition (Supplementary Fig. 3C), indicating that de novo lipogenesis is causally required for CYB561-driven mesenchymal transition. Together, these results establish CYB561 as a central regulator of metabolic reprogramming toward lipogenesis, which in turn enables key aggressive traits in BC.

### CYB561 interacts with IRE1 and activates the IRE1-XBP1-SREBF1 axis to drive lipogenic reprogramming

**Fig. 7:**
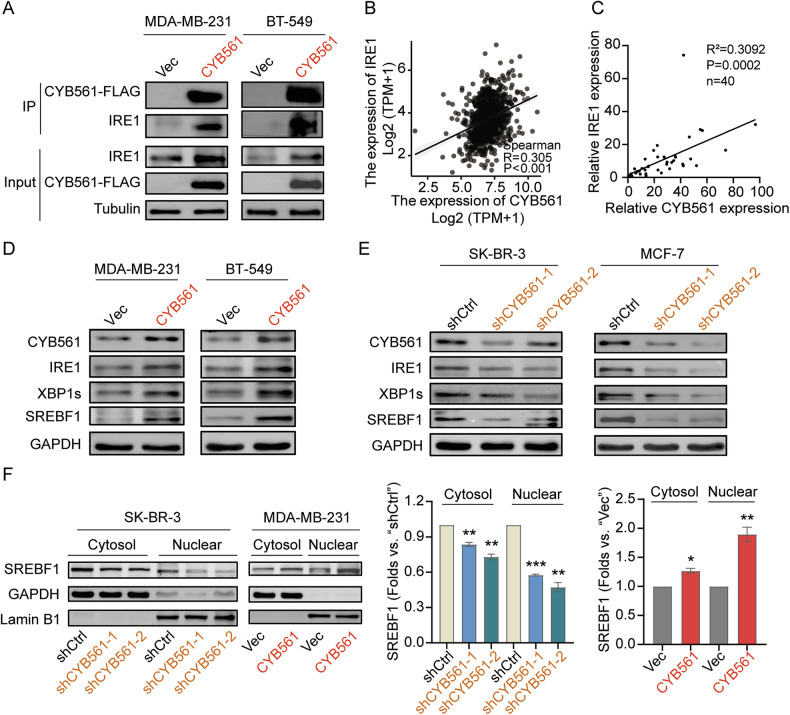
CYB561 interacts with IRE1 and activates the IRE1-XBP1-SREBF1 axis. **A** Co-immunoprecipitation (Co-IP) with anti-FLAG and followed by western blot (WB) with anti-IRE1 were performed to identify CYB561-interacting proteins in MDA-MB-231 and BT-549 cells stably transfected with FLAG-tagged CYB561 overexpression lentivirus or control virus (Vec). **B** Correlation analysis between CYB561 and IRE1 mRNA expression based on the TCGA-BRCA dataset. **C** Correlation analysis of CYB561 and IRE1 mRNA expression in 40 fresh frozen breast cancer samples. **D** WB analysis of IRE1/XBP1/SREBF1 pathway protein expression in MDA-MB-231 and BT-549 cells stably overexpressing CYB561 (CYB561 group) or empty vector (Vec group) established *via* lentiviral transduction. **E** WB analysis of IRE1/XBP1/SREBF1 pathway protein expression in SK-BR-3 and MCF-7 cells with stable CYB561 knockdown (using two distinct shRNAs: shCYB561-1 and shCYB561-2) or control (shCtrl), introduced *via* lentiviral vectors. **F** Nuclear and cytoplasmic protein fractions were isolated and subjected to WB to detect SREBF1 expression. Data were presented as mean ± standard deviation (SD, *n* = 3). * *P* < 0.05, ** *P* < 0.01, *** *P* < 0.001. Experiments in this figure were repeated three times.

To investigate how CYB561 triggers de novo lipogenesis, we performed co-immunoprecipitation followed by mass spectrometry in CYB561-overexpressing MDA-MB-231 cells and identified IRE1 as a direct binding partner of CYB561 (Fig. 7A). This interaction was further supported by positive correlations between CYB561 and IRE1 mRNA levels in both the TCGA-BRCA cohort (R^2^ = 0.305) and our 40-patient clinical cohort (R^2^ = 0.3092; Fig. 7B, C). Functionally, CYB561 overexpression increased phosphorylated IRE1, spliced XBP1 (XBP1s), and total SREBF1 protein levels, whereas CYB561 knockdown produced the opposite effects (Fig. 7D, E). Subcellular fractionation revealed that CYB561 enhanced SREBF1 abundance in both cytoplasmic and nuclear compartments (Fig. 7F), consistent with increased overall SREBF1 synthesis rather than selective promotion of nuclear import.

To determine whether SREBF1 upregulation depends on XBP1 activity, we treated CYB561-overexpressing cells with the XBP1 splicing inhibitor Toyocamycin. Pharmacological inhibition of XBP1 significantly reduced both SREBF1 mRNA and protein expression (Supplementary Fig. 4), confirming that SREBF1 induction is functionally downstream of XBP1 activation.

### CYB561 drives oncogenic phenotypes *via* the IRE1-XBP1-SREBF1 lipogenesis axis

**Fig. 8:**
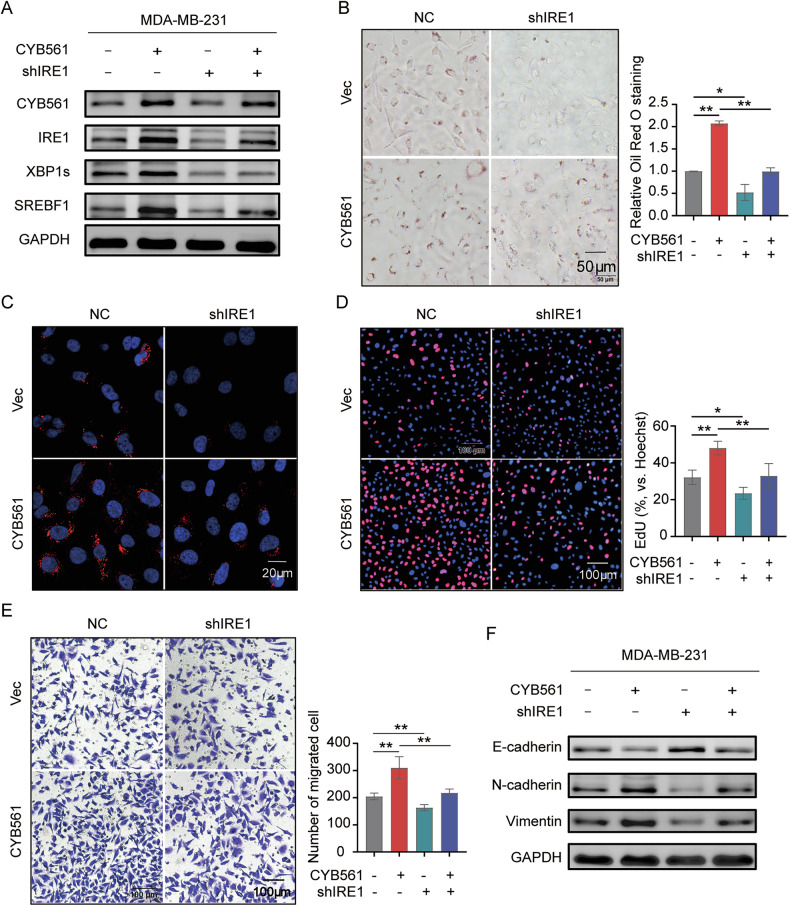
CYB561 promotes malignant phenotypes through IRE1-XBP1-SREBF1-mediated lipogenesis. CYB561-overexpressing MDA-MB-231 cells were transduced with shIRE1 or control vector for indicated durations. **A** Western blot analysis of IRE1/XBP1/SREBF1 pathway proteins. **B**, **C** Lipid droplet staining with Oil Red O (**B**) and Nile Red (**C**). **D** EdU assay for cell proliferation. **E** Transwell assay for cell migration. **F** Western blot analysis of epithelial–mesenchymal transition (EMT)-related markers. Data were presented as mean ± standard deviation (SD, *n* = 3). * *P* < 0.05, ** *P* < 0.01. Experiments in this figure were repeated three times.

To determine whether lipogenesis mediates the oncogenic effects of CYB561, we inhibited IRE1 in CYB561-overexpressing cells. IRE1 knockdown attenuated CYB561-induced upregulation of XBP1s and SREBF1 (Fig. 8A), reduced lipid droplet accumulation (Fig. 8B, C), and suppressed proliferation (Fig. 8D) and migration (Fig. 8E). IRE1 silencing also partially reversed CYB561-induced EMT, restoring E-cadherin and reducing N-cadherin and Vimentin expression (Fig. 8F). These results establish IRE1–XBP1–SREBF1 mediated lipogenesis as a key mechanism underlying CYB561’s oncogenic effects.

### CYB561 promotes oncogenic phenotypes through FAK/ERK activation and lipogenesis

**Fig. 9:**
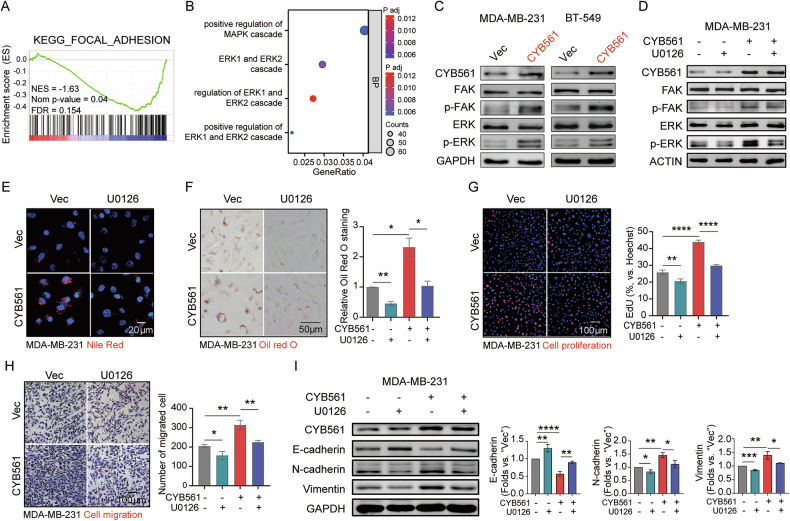
CYB561 promotes oncogenic phenotypes through FAK/ERK activation and lipogenesis. **A** GSEA and **B** GO analysis of differentially expressed genes from TCGA-BRCA cohort (CYB561^high^ vs. CYB561^low^). **C** Western blot analysis of FAK/ERK pathway proteins in MDA-MB-231 and BT-549 cells overexpressing CYB561 vs. empty vector (Vec). **D–I** CYB561-overexpressing MDA-MB-231 cells were treated with ERK inhibitor U0126 or vehicle control: **D** Western blot analysis of FAK/ERK pathway proteins. **E**, **F** Lipid droplet staining with Nile Red (**E**) and Oil Red O (**F**). **G** EdU assay for cell proliferation. **H** Transwell assay for cell migration. **I** Western blot analysis of epithelial–mesenchymal transition (EMT) markers. Data were presented as mean ± standard deviation (SD, *n* = 3). * *P* < 0.05, ** *P* < 0.01, *** *P* < 0.001, **** *P* < 0.0001. “BP” stands for biological process. “KEGG” stands for Kyoto Encyclopedia of Genes and Genomes. Experiments in this figure were repeated three times.

Focal adhesion kinase (FAK) and extracellular signal-regulated kinase (ERK) are critical signaling nodes that regulate cell proliferation, migration, survival, and remodeling of the tumor microenvironment [15, 16]. Enrichment analysis highlighted involvement of FAK and ERK signaling pathways among CYB561-associated genes (Fig. 9A, B). To determine whether CYB561’s oncogenic role depends on FAK-ERK pathway, we assessed FAK/ERK protein expression. CYB561 knockdown decreased phosphorylated FAK and ERK levels without affecting total protein expression (Supplementary Fig. 5), while overexpression increased their phosphorylation (Fig. 9C). Pharmacological inhibition of ERK by U0126 suppressed CYB561-induced ERK activation (but not FAK phosphorylation) (Fig. 9D), lipid accumulation (Fig. 9E, F), proliferation (Fig. 9G) and migration (Fig. 9H). U0126 also partially reversed EMT markers altered by CYB561 overexpression (Fig. 9I). These findings indicate that CYB561 also acts through FAK/ERK signaling to drive malignant phenotypes.

## Discussion

This study establishes a previously unrecognized role for CYB561 in BC: it functions as a central hub that coordinately drives both proliferative expansion and invasive motility—a feat that defies the classical “go-or-grow” paradigm. Traditionally, EMT is associated with cell-cycle arrest and reduced proliferation, creating a functional trade-off between migration and division [17]. However, emerging evidence demonstrates that certain oncogenic programs can uncouple this constraint by establishing hybrid EMT states or activating dual-output signaling networks that simultaneously support growth and motility [18, 19]. Our work reveals that CYB561 achieves this balance through two synergistic axes: IRE1–XBP1–SREBF1-mediated de novo lipogenesis, and FAK/ERK signaling activation.

The most salient finding of our study is the direct physical interaction between CYB561 and the ER stress sensor IRE1, which initiates a potent lipogenic cascade. CYB561 overexpression enhances IRE1 phosphorylation, XBP1 splicing, and subsequent upregulation of SREBF1—a master transcriptional regulator of fatty acid and cholesterol biosynthesis. Importantly, pharmacological inhibition of XBP1 splicing abrogates SREBF1 induction, confirming a functional dependency. Subcellular fractionation further shows that SREBF1 accumulates in both cytoplasmic and nuclear compartments, consistent with increased precursor synthesis rather than altered nuclear import—a mechanism aligned with canonical SREBF1 regulation *via* sterol-dependent proteolysis [20]. Supporting causality, we demonstrate that FASN inhibition reverses CYB561-driven EMT, while exogenous palmitic acid rescues mesenchymal marker expression, establishing de novo lipogenesis as a necessary and partially sufficient driver of EMT.

Concurrently, CYB561 activates the FAK/ERK pathway, as evidenced by increased phosphorylation of FAK and ERK. Notably, ERK is a well-documented integrator of proliferation and migration signals: it promotes G1/S progression via cyclin D1 upregulation while also phosphorylating EMT transcription factors (e.g., Slug, Twist) to enhance invasiveness [21, 22]. Thus, the FAK/ERK axis provides a direct molecular bridge between CYB561-induced growth and motility. Critically, these two pathways likely reinforce each other: SREBF1-driven lipid remodeling may facilitate growth factor receptor trafficking and membrane raft organization, thereby potentiating FAK/ERK activation; conversely, ERK can phosphorylate and stabilize SREBF1, creating a feed-forward loop that sustains both metabolic reprogramming and a “go-and-grow” phenotype [23, 24].

An important consideration arising from our work is the potential contribution of CYB561’s canonical redox and iron-metabolizing functions to the observed phenotypes. CYB561 is a well-established ascorbate-dependent ferrireductase that reduces Fe³⁺ to Fe²⁺ to support non-transferrin-bound iron uptake and vitamin C recycling [25]. Given that iron and reactive oxygen species (ROS) are potent modulators of ER stress, lipid peroxidation, and ferroptosis—all of which intersect with lipid metabolism and EMT [26]—it is plausible that CYB561’s enzymatic activity indirectly influences the IRE1–XBP1–SREBF1 axis. For instance, iron overload exacerbates ER stress and activates IRE1α in alcohol- and obesity-induced liver injury [27], while ROS generated *via* Fenton chemistry can promote SREBF1 cleavage through oxidation of INSIG proteins [28]. Moreover, lipid peroxidation—a hallmark of ferroptosis—has been linked to metastatic plasticity in BC, where partial resistance to ferroptosis enables survival during dissemination [29, 30]. Thus, CYB561 could potentially couple its ferrireductase activity to lipogenic reprogramming not only through direct protein–protein interaction with IRE1 (as demonstrated here) but also *via* modulation of the intracellular iron–redox landscape.

Notably, whether the enzymatic activity of CYB561 is required for IRE1 activation and downstream oncogenic effects remains an open question. While our data establish a physical and functional link between CYB561 and IRE1, they do not distinguish whether this interaction depends on CYB561’s redox function or represents a scaffolding role independent of catalysis. Future studies employing catalytically inactive CYB561 mutants will be essential to dissect these mechanisms. This represents a key limitation of the present study and a critical direction for future work.

Furthermore, our findings raise a deeper question: does CYB561-driven lipid metabolism extend beyond cell-autonomous functions to shape the tumor microenvironment? Tumor-derived lipids can suppress T cell function or polarize macrophages toward an immunosuppressive M2 phenotype [31, 32]. Whether CYB561-overexpressing tumor cells exploit similar mechanisms to create an immune-evasive niche warrants investigation.

From a therapeutic perspective, our model suggests that co-targeting metabolic and signaling vulnerabilities may yield synergistic efficacy. In TNBC, combining a future CYB561 inhibitor with FASN or SREBF1 blockade could dismantle the lipogenic engine driving both proliferation and EMT. In HER2-positive BC, combining CYB561 targeting with trastuzumab and an ERK inhibitor might constitute a multi-pronged precision therapy strategy, potentially preventing the emergence of resistance.

Despite these advances, several key questions remain. For example, the structural basis of the CYB561-IRE1 interaction is still unclear. Determining the three-dimensional structure of their binding interface is a prerequisite for designing specific blocking peptides or small molecules. In addition, understanding the role of specific lipid species (e.g., phosphatidylinositols, sphingolipids) as signaling mediators in this axis is pivotal for deciphering its underlying functional mechanism. Moreover, investigating whether CYB561 expression itself is regulated by upstream signals represents another valuable area of exploration, which would help us understand the complete regulatory network of this pathway.

**Fig. 10:**
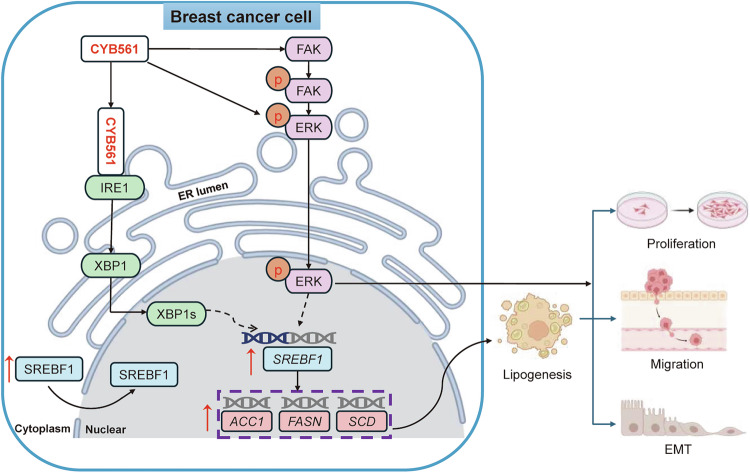
Schematic diagram of the mechanism by which CYB561 regulates breast cancer progression. In breast cancer, highly expressed CYB561 promotes tumor progression by interacting with IRE1 to activate the IRE1/XBP1/SREBF1 axis, while concurrently stimulating the FAK/ERK signaling pathway. These mechanisms collectively drive lipid metabolic reprogramming, enhance tumor cell survival, proliferation, migration, invasion, and epithelial–mesenchymal transition.

In summary, this study redefines CYB561 not merely as a signaling participant but as a core metabolic orchestrator that integrates ER stress, lipogenesis, and kinase signaling to enable a rare “go-and-grow” phenotype in aggressive BC (Fig. 10). The elucidation of this multifaceted role provides a foundational framework for novel combination therapies targeting refractory subtypes.

## Materials and methods

### Ethics statement

Written informed consent was obtained from all participants prior to sample collection. All human samples were stored in the Biobank of Affiliated Kunshan Hospital of Jiangsu University. The study was approved by the Ethics Committee of Affiliated Kunshan Hospital of Jiangsu University (Ethics No. 2024-03-063-H00-K01). All animal procedures were performed in compliance with guidelines established by the hospital’s Institutional Animal Care and Use Committee.

### Reagents

Short hairpin RNAs (shRNAs) targeting CYB561 and IRE1, along with a scrambled shRNA control, were procured from Shanghai GeneChem Co., Ltd (Shanghai, China). Lentivirus for CYB561 overexpression and corresponding control vectors were obtained from Suzhou GENEWIZ Biotechnology Co., Ltd (Suzhou, China). U0126-EtOH (HY-12031), Nile Red (HY-D0718), Toyocamycin (HY-103248), C75 (HY-12364) and Palmitic acid (HY-N0830) were purchased from MedChemexpress Co. Ltd (NJ, USA) and dissolved in DMSO to a stock concentration of 10 mM.

### Patients and clinical specimens

Forty pairs of BC and para-tumor normal tissues were acquired from the Biobank of Affiliated Kunshan Hospital of Jiangsu University for quantitative PCR analysis. All cases were histopathologically confirmed as primary BC.

### Cell culture and viral transduction

The human BC cell lines MDA-MB-231 (Cat# ZQ0118, TNBC cell line), BT-549 (Cat# CX0068, TNBC cell line), MCF-7 (Cat# ZQ0071, Luminal type BC cell line), and SK-BR-3 (Cat# ZQ0080, HER2^+^ BC cell line), along with the non-tumorigenic breast epithelial cell line MCF-10A (Cat# CL-0525), were acquired from Shanghai Zhong Qiao Xin Zhou Biotechnology Co., Ltd (Shanghai, China), Boster Biological Technology, Ltd (Wuhan, China) and Procell Life Science & Technology Co., Ltd (Wuhan, China), respectively, and maintained under standard conditions as previously described [33]. All cell lines were authenticated *via* short tandem repeat (STR) profiling within the last 3 years and confirmed mycoplasma-free.

Transduction with shRNA or overexpression constructs was carried out following manufacturer’s instructions, followed by selected with puromycin. The shRNA sequences used were as follows: sh-CYB561#1, GGGCAAGTATAGCGCATTTGA; sh-CYB561#2, GGCTTCAGCTTCTTCCTGTTC; sh-IRE1#1, GGCGCATCACAAAGTGGAAGT; sh-IRE1#2, GCTCAACTACTTGAGGAATTA; Scrambled RNA control, TTCTCCGAACGTGTCACGT.

### Bioinformatic analysis

RNA-seq data from the TCGA-BRCA cohort were retrieved and analyzed. Based on median CYB561 expression, tumor samples were categorized into high- and low-expression groups. Differential gene expression analysis was performed, followed by Gene Ontology (GO) and Gene Set Enrichment Analysis (GSEA) to identify pathways associated with CYB561 expression.

### Reverse transcription-quantitative polymerase chain reaction (RT-qPCR)

Total RNA was extracted using established methods. qRT-PCR was performed with gene-specific primers synthesized by Suzhou GENEWIZ Biotechnology Co., Ltd. The sequences are as follows: *CYB561*-forward, 5’-GTCTTCAGGAACGAAGCTAAAC-3’, *CYB561*-reverse, 5’-CTTCTTCCTGTGGTAGTCGAAC-3’; *β-actin*-forward, 5’-TCCTTCCTGGGCATGGAGTC-3’, *β-actin*-reverse, 5’-GTAACGCAACTAAGTCATAGTC-3’; *SREBF1*-forward, 5’-ACTTCTGGAGGCATCGCAAGCA-3’, *SREBF1*-reverse, 5’-AGGTTCCAGAGGAGGCTACAAG-3’; *ACC1*-forward, 5’-TCACACCTGAAGACCTTAAAGCC-3’, *ACC1*-reverse 5’-AGCCCACACTGCTTGTACTG-3’; *FASN*-forward, 5’-ACAGCGGGGAATGGGTACT-3’, *FASN*-reverse 5’-GACTGGTACAACGAGCGGAT-3’; *SCD*-forward, 5’-CACTTGGGAGCCCTGTATGG-3’, *SCD*-reverse 5’-TGAGCTCCTGCTGTTATGCC-3’; *HMGCS*-forward, 5’-GTTGGCGGCTATAAAGCTGGT-3’, *HMGCS*-reverse 5’-CCTTCGGGCACAAGCG-3’; *HMGCR*-forward, 5’-GTGAGATCTGGAGGATCCAAGG-3’, *HMGCR*-reverse 5’-GATGGGAGGCCACAAAGAGG-3’; *IRE1*-forward, 5’-AGTATGTGGAGCAGAAGGAC-3’, *IRE1*-reverse 5’-GTTGTGTGGCTTTAGGTCTC-3’.

### Western blotting

Cells were lysed in RIPA buffer supplemented with phosphatase and protease inhibitors at 4 °C. Proteins were separated on 10% SDS-polyacrylamide gels and transferred to polyvinylidine difluoride (PVDF) membranes (Merck Millipore, Sigma-Aldrich (Shanghai) Trading Co., Ltd.). After blocking with 5% non-fat milk in TBST for 1 h at 37 °C, membranes were incubated overnight at 4 °C with primary antibodies. In which, Anti-CYB561 (Cat# DF8938), anti-β-actin (Cat# AF7018), anti-N-cadherin (Cat# AF5239), anti-Vimentin (Cat# AF7013), anti-FAK (Cat# AF6397), anti-phospho-FAK (Tyr397, Cat# AF3398), GAPDH (Cat# AF7021) and anti-β-tubulin (Cat# AF7011) are the products of Affinity Biosciences (Jiangsu, China); Anti-E-cadherin (Cat# 20874-1-AP), anti-IRE1 (Cat# 27528-1-AP), anti-XBP1 (Cat# 24868-1-AP) and anti-SREBF1 (Cat# 14088-1-AP) are purchased from Proteintech Group, Inc. (Rosenont, USA); Anti-phospho-p44/42 MAPK (ERK1/2) (Cat# 4370) is the product of Cell Signaling Technology (Shanghai) Biological Reagent Co., Ltd. (Shanghai, China); Anti-ERK1/2 (Cat# sc-514302) is acquired from Santa Cruz Biotechnology (Shanghai) Co., Ltd. (Shanghai, China); Anti-Lamin B1 (Cat# A16909) is purchased from ABclonal Biotech Co., Ltd. (Wuhan, China). HRP-conjugated secondary antibodies were applied for 2 h at room temperature, and signals were detected using an ECL Enhanced kit (Cat# RM00021; ABclonal Biotech Co., Ltd., Wuhan, China) on a Touch imaging system (e-Blot).

### Immunohistochemistry (IHC) and scoring

Tumor tissues (5 μm) were prepared from paraffin-embedded samples fixed in 4% paraformaldehyde. IHC was performed using anti-CYB561 (Cat# HPA014753, Atlas Antibodies, Stockholm Sweden) and anti-Ki-67 antibodies (Cat# ab15580; Abcam Trading (Shanghai) Co., Ltd.) according to standard protocols. Staining was evaluated independently by two pathologists using a semi-quantitative scoring system based on staining intensity and the percentage of positive cells across five high-power fields. Staining intensity scoring: unstained (0 points), pale yellow (1 point), brownish-yellow (2 points), tan brown (3 points). Positive cell percentage scoring: ≤5% positive cells (0 points), 6–25% (1 point), 26–50% (2 points), ≥51% (3 points). The final score was calculated by summing both subscores, with interpretation as follows: <3 points = negative (-); ≥3 points = positive (+); ≥4 points = weakly positive (++); ≥6 points = strongly positive (+++).

### Cell proliferation assay

Cell viability was assessed using Cell Counting Kit-8 (CCK-8) and 5-ethynyl-2′- deoxyuridine (EdU) assays. Cells were seeded in 96-well plates at a density of 2,000 cells/well in DMEM supplemented with 10% FBS. CCK-8 reagent (Cat# BS350B, Hefei Biosharp Biotechnology Co., Ltd., Hefei, China) was added and incubated for 2 h once a day for 4 or 5 consecutive days. EdU staining was conducted using an EdU kit (Cat# C10310-1; Guangzhou RiboBio Co. Ltd., Guangzhou, China) following the manufacturer’s instructions.

### Cell migration and invasion assay

Cell migration and invasion were evaluated using transwell chambers (Corning Inc., NY, USA) with 8 µm pore membranes. For invasion assays, chambers were pre-coated with Matrigel (BD Biosciences, CA, USA). Procedures were performed as previously described [33].

### Xenograft tumor model

Animal protocols have been approved by Institutional Animal Care and Use Committee (IACUC) and the Ethics Review Board of The Affiliated Kunshan Hospital of Jiangsu University. SK-BR-3 cells (1 × 10^6^) transduced with CYB561-shRNA or scramble shRNA control were resuspended in a PBS/Matrigel mixture (3:2 ratio) and subcutaneously injected into the bilateral inguinal regions of female BALB/c nude mice (4–5 weeks old), which were obtained from Kunshan Mingqian Microbiological Research Institute Co., Ltd. (Kunshan, Jiangsu, China). Tumor dimensions and mouse body weights were monitored regularly. Tumor volume was calculated as length (mm) × width^2^ (mm)/2. Mice were humanely euthanized when the largest tumor reached acceptable size limits. Resected tissues were imaged and stored at −80 °C.

### Co-immunoprecipitation and mass spectrometry

MDA-MB-231 cells (1 × 10^7^) stably expressing CYB561-FLAG or transfected with empty vector were lysed, and proteins (2000 μg) were immunoprecipitated with 14 μl anti-Flag (Cat# 14793, Cell Signaling Technology) at 4 °C overnight, followed by incubation with 40 μl Pierce Protein A/G magnetic beads (Cat# 88802, Thermo Scientific Technology (China) Co., Ltd., Shanghai, China) at 4 °C for 6 h. Beads were washed, then were subjected to analysis by mass spectrometry or western blotting.

### Subcellular fractionation

The cells are harvested, lysis and proteins extracted with Nuclear and Cytoplasmic Protein Extraction Kit (Cat# P0027, Beyotime Biotechnology, Shanghai, China) according to the manufacturer’s recommendations. Briefly, adherent cells were scraped, and the cell pellet was obtained by centrifugation. Next, resuspend cell pellet in 100 µL of cytoplasmic protein extraction reagent A per 10^6^ cells, and vortex vigorously for 5 s. Incubate on ice for 10 min, then add 10 µL of cytoplasmic protein extraction reagent B. Vortex vigorously for 5 s and centrifuge the preparation for 5 min at 12,000 × g at 4 °C. Carefully remove the cytoplasmic extract from the nuclear pellet. Add 50 µL of nuclear protein extraction reagent to nuclear pellet and vortex for 15 s at the maximum speed to suspend the precipitation. Incubate the extract on ice for 30 min with vortex (15 s) every 2 min. Centrifuge the suspension for 10 min at 14,000 × g at 4 °C and transfer the supernatant into a pre cooled microcentrifuge vial.

### Oil Red O staining

Oil Red O staining was performed with Oil Red O Kit (Cat# C0158S, Beyotime Biotechnology, Shanghai, China). Cells were washed with PBS once, and fixed with 4% paraformaldehyde for 10 min. The cells were washed twice with PBS and added an appropriate amount of staining detergent to cover for 20 s. Remove the staining detergent and stain with the newly prepared oil red O staining solution for 20 min. Wash the cells with staining detergent for 30 s and PBS for 20 s. Cover the cells evenly with PBS. The pictures were taken by an Olympus IX73 microscope.

### Nile Red staining

After seeding cells on the 8-chamber slide, the cells were subjected to fixation employing a 4% paraformaldehyde solution for a duration of 15 min. Subsequently, they were stained with a concentration of 200 nM Nile Red (Cat# HY-D0718, MedChemexpress Co. Ltd, NJ, USA) for a period of 30 min in order to see the lipid droplets. Subsequently, the nucleus was stained with 4’,6-diamidino-2-phenylindole (DAPI, Cat# BL105A, Biosharp) as a counterstain. The acquisition of images was conducted employing a laser scanning confocal microscope (LSM 900, Carl Zeiss Microscopy GmbH, Jena, Germany).

### Statistical analysis

In vitro experiments were repeated three times. Data are presented as mean ± standard deviation. Comparisons between two groups were performed using an independent Student’s t-test. Comparisons among multiple groups were analyzed by one-way analysis of variance (ANOVA). The relationship between CYB561 and IRE1 mRNA levels was assessed using Spearman’s rank correlation coefficient. A two-tailed *p*-value of less than 0.05 was considered statistically significant.

## Supplementary information

Supplementary Figures

Original western blot images

## Data Availability

All data and materials are available upon request by contacting the corresponding author.

## References

[1] Bray F, Laversanne M, Sung H, Ferlay J, Siegel RL, Soerjomataram I, et al. Global cancer statistics 2022: GLOBOCAN estimates of incidence and mortality worldwide for 36 cancers in 185 countries. CA Cancer J Clin. 2024;74:229–63. 38572751 10.3322/caac.21834

[2] Bianchini G, De Angelis C, Licata L, Gianni L. Treatment landscape of triple-negative breast cancer - expanded options, evolving needs. Nat Rev Clin Oncol. 2022;19:91–113. 34754128 10.1038/s41571-021-00565-2

[3] Xiong X, Zheng LW, Ding Y, Chen YF, Cai YW, Wang LP, et al. Breast cancer: pathogenesis and treatments. Signal Transduct Target Ther. 2025;10:49. 39966355 10.1038/s41392-024-02108-4 PMC11836418

[4] Marin A, Mamun AA, Patel H, Akamatsu H, Ye D, Sudhan DR, et al. Acquired secondary HER2 mutations enhance HER2/MAPK signaling and promote resistance to HER2 kinase inhibition in breast cancer. Cancer Res. 2023;83:3145–58. 37404061 10.1158/0008-5472.CAN-22-3617 PMC10530374

[5] Rohrig F, Schulze A. The multifaceted roles of fatty acid synthesis in cancer. Nat Rev Cancer. 2016;16:732–49. 27658529 10.1038/nrc.2016.89

[6] Menendez JA, Lupu R. Fatty acid synthase and the lipogenic phenotype in cancer pathogenesis. Nat Rev Cancer. 2007;7:763–77. 17882277 10.1038/nrc2222

[7] Ferraro GB, Ali A, Luengo A, Kodack DP, Deik A, Abbott KL, et al. Fatty acid synthesis is required for breast cancer brain metastasis. Nat Cancer. 2021;2:414–28. 34179825 10.1038/s43018-021-00183-y PMC8223728

[8] Gruslova A, McClellan B, Balinda HU, Viswanadhapalli S, Alers V, Sareddy GR, et al. FASN inhibition as a potential treatment for endocrine-resistant breast cancer. Breast Cancer Res Treat. 2021;187:375–86. 33893909 10.1007/s10549-021-06231-6

[9] Sen U, Coleman C, Sen T. Stearoyl coenzyme A desaturase-1: multitasker in cancer, metabolism, and ferroptosis. Trends Cancer. 2023;9:480–9. 37029018 10.1016/j.trecan.2023.03.003

[10] Yang X, Zhao Y, Shao Q, Jiang G. Cytochrome b561 serves as a potential prognostic biomarker and target for breast cancer. Int J Gen Med. 2021;14:10447–64. 35002301 10.2147/IJGM.S338878 PMC8722309

[11] Zhou X, Shen G, Ren D, Guo X, Han J, Guo Q, et al. Expression and clinical prognostic value of CYB561 in breast cancer. J Cancer Res Clin Oncol. 2022;148:1879–92. 35486183 10.1007/s00432-022-03928-z PMC11800974

[12] Zhou X, Guo X, Han J, Wang M, Liu Z, Ren D, et al. Cytochrome b561 regulates iron metabolism by activating the Akt/mTOR pathway to promote breast cancer cells proliferation. Exp Cell Res. 2023;431:113760. 37634562 10.1016/j.yexcr.2023.113760

[13] Zhao T, Wang C, Zhao N, Qiao G, Hua J, Meng D, et al. CYB561 promotes HER2+ breast cancer proliferation by inhibiting H2AFY degradation. Cell Death Discov. 2024;10:38. 38245506 10.1038/s41420-024-01804-y PMC10799939

[14] Cui Q, Josephraj S, Gu B, Liu JY, Zhang JT. Targeting fatty acid synthase for cancer drug discovery: Retrospective analyses and outlook. Pharmacol Rev. 2026;78:100105. 41519050 10.1016/j.pharmr.2025.100105 PMC13169002

[15] Zhang J, Hochwald SN. The role of FAK in tumor metabolism and therapy. Pharmacol Ther. 2014;142:154–63. 24333503 10.1016/j.pharmthera.2013.12.003 PMC6349254

[16] Lavoie H, Gagnon J, Therrien M. ERK signalling: a master regulator of cell behaviour, life and fate. Nat Rev Mol Cell Biol. 2020;21:607–32. 32576977 10.1038/s41580-020-0255-7

[17] Hatzikirou H, Basanta D, Simon M, Schaller K, Deutsch A. Go or grow’: the key to the emergence of invasion in tumour progression? Math Med Biol. 2012;29:49–65. 20610469 10.1093/imammb/dqq011

[18] Nieto MA, Huang RY, Jackson RA, Thiery JP. EMT: 2016. Cell. 2016;166:21–45. 27368099 10.1016/j.cell.2016.06.028

[19] Kroger C, Afeyan A, Mraz J, Eaton EN, Reinhardt F, Khodor YL, et al. Acquisition of a hybrid E/M state is essential for tumorigenicity of basal breast cancer cells. Proc Natl Acad Sci USA. 2019;116:7353–62. 30910979 10.1073/pnas.1812876116 PMC6462070

[20] Wang X, Sato R, Brown MS, Hua X, Goldstein JL. SREBP-1, a membrane-bound transcription factor released by sterol-regulated proteolysis. Cell. 1994;77:53–62. 8156598 10.1016/0092-8674(94)90234-8

[21] Samson SC, Khan AM, Mendoza MC. ERK signaling for cell migration and invasion. Front Mol Biosci. 2022;9:998475. 36262472 10.3389/fmolb.2022.998475 PMC9573968

[22] Montserrat N, Mozos A, Llobet D, Dolcet X, Pons C, de Herreros AG, et al. Epithelial to mesenchymal transition in early stage endometrioid endometrial carcinoma. Hum Pathol. 2012;43:632–43. 21940036 10.1016/j.humpath.2011.06.021

[23] Fuhrman B, Nitzan O, Karry R, Volkova N, Dumler I, Aviram M. Urokinase plasminogen activator (uPA) stimulates cholesterol biosynthesis in macrophages through activation of SREBP-1 in a PI3-kinase and MEK-dependent manner. Atherosclerosis. 2007;195:e108–16. 17681345 10.1016/j.atherosclerosis.2007.06.025

[24] Boonsong T, Norton L, Chokkalingam K, Jewell K, Macdonald I, Bennett A, et al. Effect of exercise and insulin on SREBP-1c expression in human skeletal muscle: potential roles for the ERK1/2 and Akt signalling pathways. Biochem Soc Trans. 2007;35:1310–1. 17956338 10.1042/BST0351310

[25] Zhou X, An Z, Lei H, Liao H, Guo X. Role of the human cytochrome b561 family in iron metabolism and tumors (Review). Oncol Lett. 2025;29:111. 39802312 10.3892/ol.2024.14857 PMC11718626

[26] Guo Z, Chi R, Peng Y, Sun K, Liu H, Guo F, et al. The role and interactive mechanism of endoplasmic reticulum stress and ferroptosis in musculoskeletal disorders. Biomolecules. 2024;14:1369. 10.3390/biom14111369 PMC11591632 39595546

[27] Tan TC, Crawford DH, Jaskowski LA, Subramaniam VN, Clouston AD, Crane DI, et al. Excess iron modulates endoplasmic reticulum stress-associated pathways in a mouse model of alcohol and high-fat diet-induced liver injury. Lab Invest. 2013;93:1295–312. 24126888 10.1038/labinvest.2013.121

[28] Zeng H, Qin H, Liao M, Zheng E, Luo X, Xiao A, et al. CD36 promotes de novo lipogenesis in hepatocytes through INSIG2-dependent SREBP1 processing. Mol Metab. 2022;57:101428. 34974159 10.1016/j.molmet.2021.101428 PMC8810570

[29] Park MN, Choi J, Ribeiro R, Delfino DV, Ko SG, Kim B. The redox-adhesion-exosome (RAX) hub in cancer: lipid peroxidation-driven EMT plasticity and ferroptosis defense with HNE/MDA signaling and lipidomic perspectives. Antioxidants. 2025;14:1474. 10.3390/antiox14121474 PMC12729738 41462674

[30] Guo F, Zong S, Zhang X, Ren Z, Shao H, Li J, et al. Ferroptosis and metastasis: molecular checkpoints, microenvironmental dynamics, and therapeutic opportunities. Mol Cancer. 2026;25:45. 10.1186/s12943-025-02544-y PMC12947519 41535945

[31] Zhu Y, Zhou Z, Du X, Lin X, Liang ZM, Chen S, et al. Cancer cell-derived arginine fuels polyamine biosynthesis in tumor-associated macrophages to promote immune evasion. Cancer Cell. 2025;43:1045–60. 40185095 10.1016/j.ccell.2025.03.015

[32] Yang J, Yu X, Xiao M, Xu H, Tan Z, Lei Y, et al. Histone lactylation-driven feedback loop modulates cholesterol-linked immunosuppression in pancreatic cancer. Gut. 2025;74:1859–72. 40467104 10.1136/gutjnl-2024-334361 PMC12573332

[33] Yang X, Tao Y, Xu Y, Cai W, Shao Q. SLC35A2 expression drives breast cancer progression via ERK pathway activation. FEBS J. 2024;291:1483–505. 38143314 10.1111/febs.17044

