# Supplementary material for: Oncoprotein CYB561, acting in IRE1-XBP1-SREBF1 and FAK-ERK pathway, promotes breast cancer lipogenesis and progression
Source: Cell Death Discov. 2026 Apr 13;12:227. doi: 10.1038/s41420-026-03101-2 (PMC13184095; doi:10.1038/s41420-026-03101-2)
Supplement: Supplementary file 1 — Supplementary Figures [file 41420_2026_3101_MOESM1_ESM.pdf]

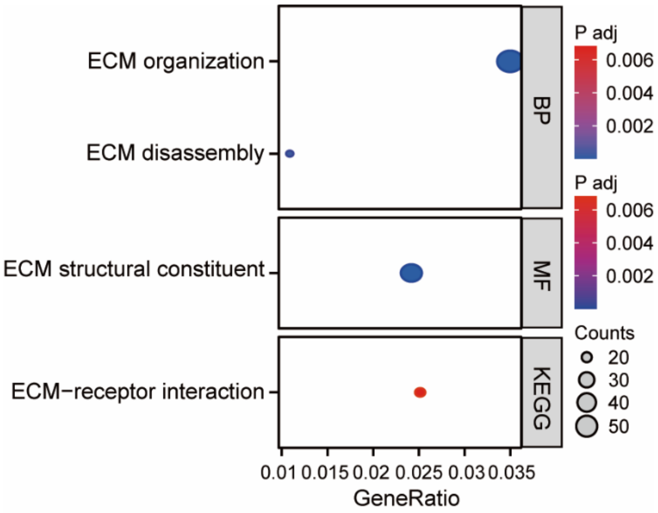

Supplementary Fig.1 Functional enrichment analysis of differentially expressed genes in CYB561^{high} versus CYB561^{low} breast cancer subgroups (TCGA-BRCA cohort). GO and KEGG analyses of differentially expressed genes between CYB561^{high} and CYB561^{low} expression groups in the TCGA-BRCA cohort. “ECM” stands for extracellular matrix. “BP” stands for biological process. “MF” stands for molecular function. “KEGG” stands for Kyoto Encyclopedia of Genes and Genomes.

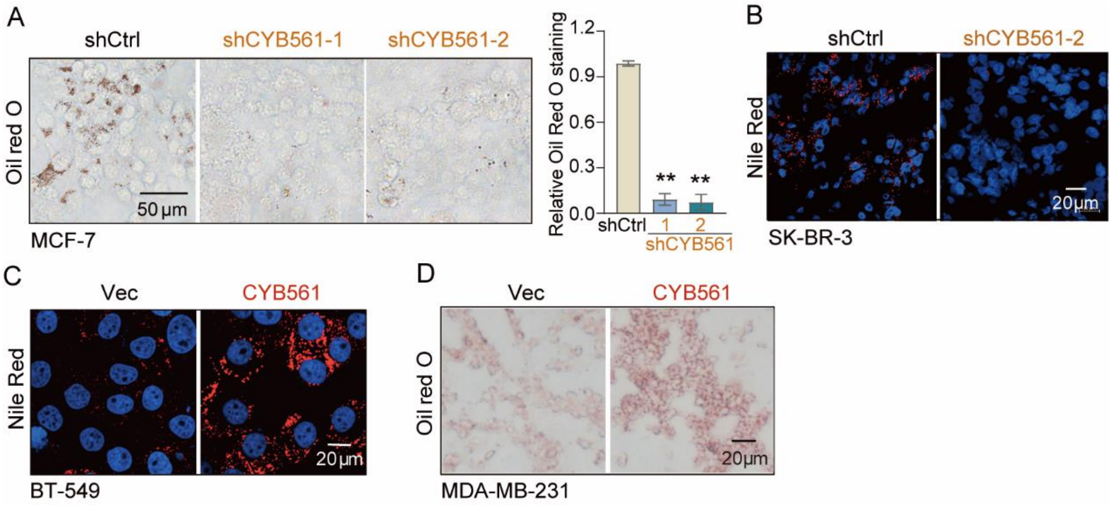

Supplementary Fig.2 CYB561 drives *de novo* lipogenesis in breast cancer cells. (A) Lipid droplet content was assessed by Oil Red O staining in MCF-7 cells with stable CYB561 knockdown (using two distinct shRNAs: shCYB561-1 and shCYB561-2) or control (shCtrl), established *via* lentiviral vectors. (B) Frozen sections of tumor tissues from subcutaneous xenograft models in nude mice were stained with Nile Red to evaluate lipid droplet content in CYB561-knockdown tumors. (C) Lipid droplet content was detected by Nile Red staining in BT-549 cells stably overexpressing CYB561 (CYB561 group) or empty vector (Vec group) *via* lentiviral transduction. (D) Frozen sections of tumor tissues from subcutaneous xenograft models were subjected to Oil Red O staining to analyze lipid droplet content in CYB561-overexpressing tumors. Data were presented as mean ± standard deviation (SD, n=3). ** $P < 0.01$. Experiments in this figure were repeated three times.

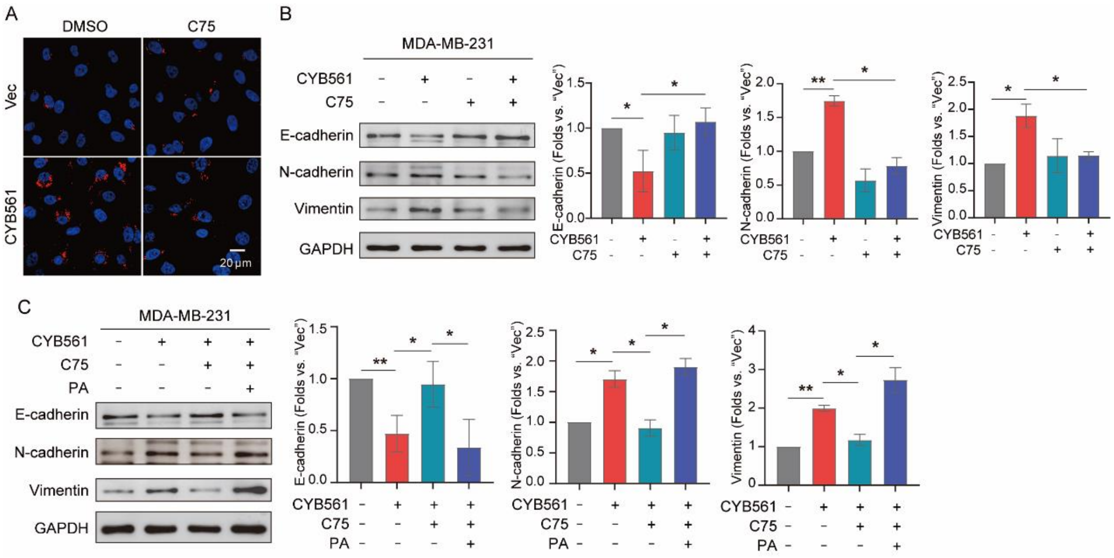

Supplementary Fig.3 *De novo* lipogenesis is essential for CYB561-driven epithelial-mesenchymal transition in breast cancer cells. CYB561-overexpressing breast cancer cells were treated with the FASN inhibitor C75. (A) Lipid droplet staining with Nile Red. (B) Western blot analysis of key epithelial-mesenchymal transition (EMT) proteins. CYB561-overexpressing breast cancer cells were treated with the FASN inhibitor C75 and palmitic acid (PA). (C) Western blot analysis of EMT-related markers. Data were presented as mean ± standard deviation (SD, n=3). * $P < 0.05$, ** $P < 0.01$. Experiments in this figure were repeated three times.

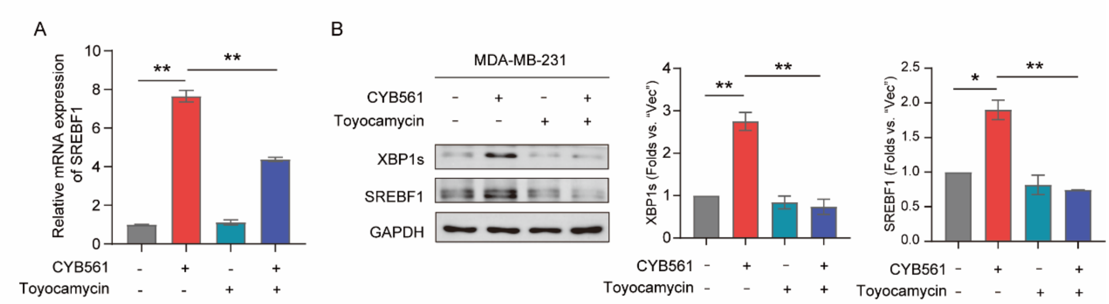

Supplementary Fig. 4 SREBF1 upregulation by CYB561 is dependent on XBP1 splicing activity. CYB561-overexpressing breast cancer cells were treated with the XBP1 splicing inhibitor Toyocamycin. Rt-qPCR (A) and Western blot (B) analyses of *SREBF1* mRNA and protein levels were conducted. Data were presented as mean ± standard deviation (SD, n=3). * $P < 0.05$, ** $P < 0.01$. Experiments in this figure were repeated three times.

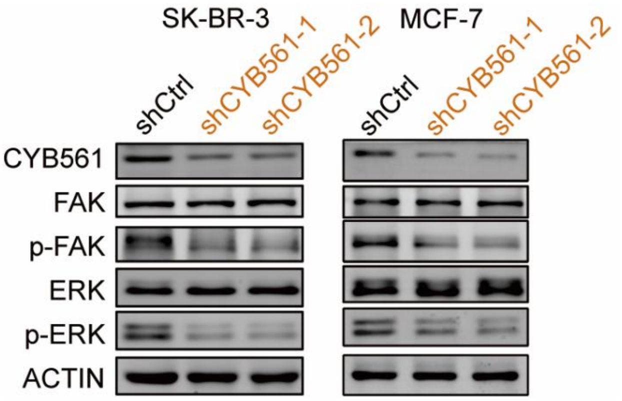

Supplementary Fig. 5 CYB561 knockdown inhibits FAK/ERK activation. Western blot analysis of FAK/ERK pathway proteins in SK-BR-3 and MCF-7 cells with CYB561 knockdown (shCYB561-1/2) vs. control (shCtrl). Experiments in this figure were repeated three times.
