# Supplementary material for: Oncoprotein CYB561, acting in IRE1-XBP1-SREBF1 and FAK-ERK pathway, promotes breast cancer lipogenesis and progression
Source: Cell Death Discov. 2026 Apr 13;12:227. doi: 10.1038/s41420-026-03101-2 (PMC13184095; doi:10.1038/s41420-026-03101-2)

Fig.2A

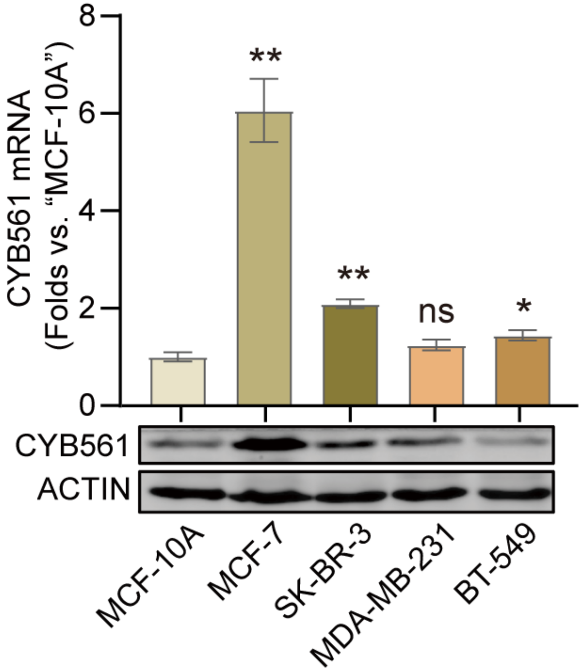

CYB561 mRNA (Folds vs. "MCF-10A")
8
6
4
2
0
**
**
ns
*
CYB561
ACTIN
MCF-10A
MCF-7
SK-BR-3
MDA-MB-231
BT-549

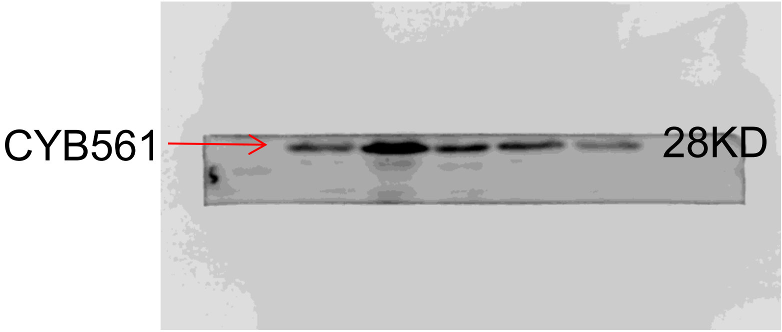

CYB561
28KD

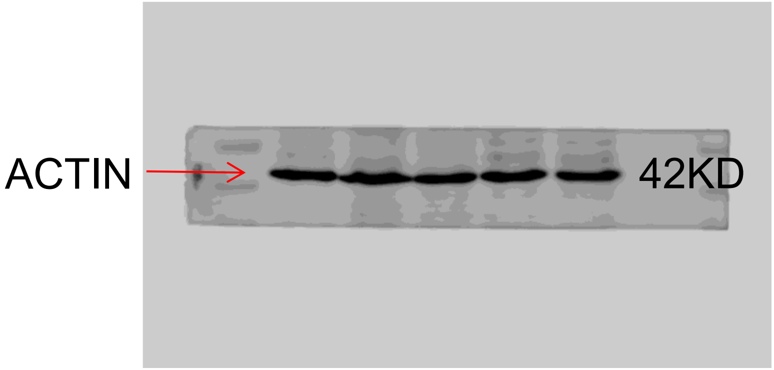

ACTIN
42KD

Fig.2B

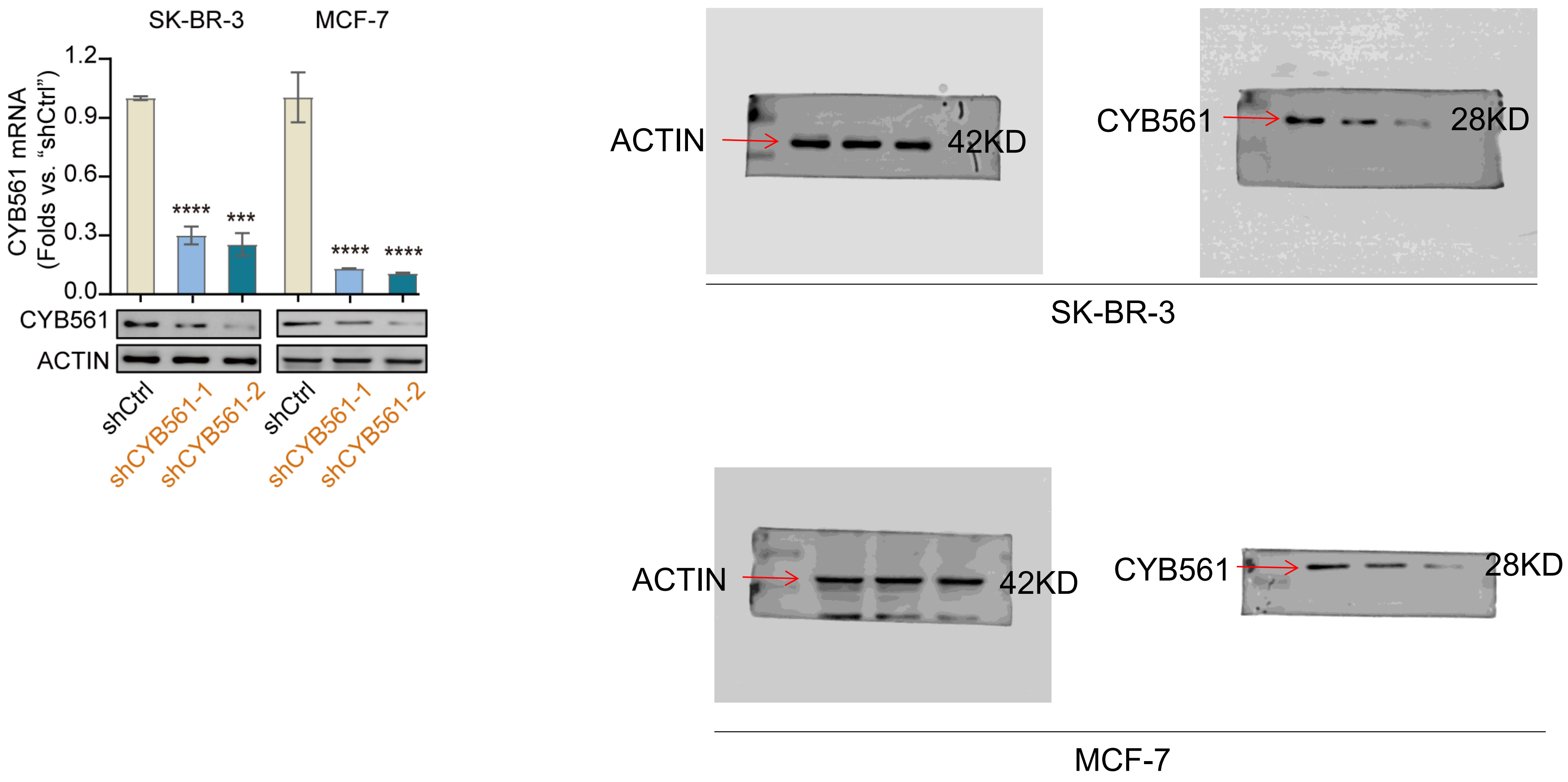
SK-BR-3
MCF-7
CYB561 mRNA
(Folds vs. "shCtrl")
1.2
0.9
0.6
0.3
0.0
****
***
****
****
CYB561
ACTIN
shCtrl
shCYB561-1
shCYB561-2
shCtrl
shCYB561-1
shCYB561-2
ACTIN
42KD
CYB561
28KD
SK-BR-3
ACTIN
42KD
CYB561
28KD
MCF-7

Fig.2C

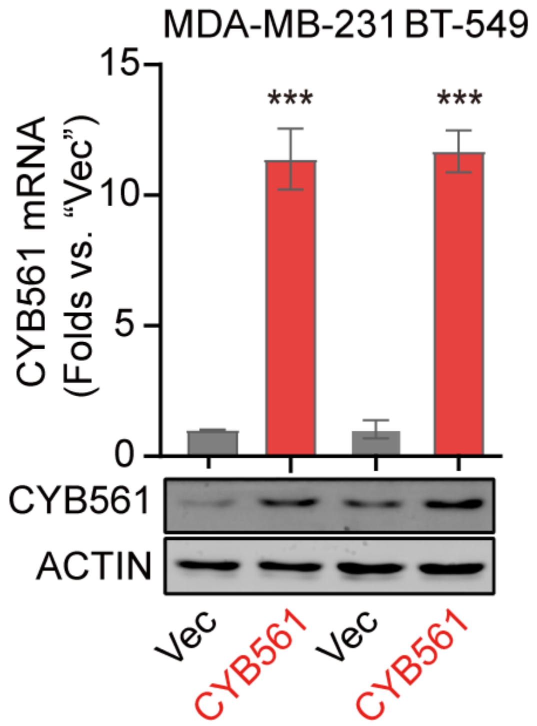
MDA-MB-231
BT-549
CYB561 mRNA (Folds vs. "Vec")
15
10
5
0
***
***
CYB561
ACTIN
Vec
CYB561
Vec
CYB561

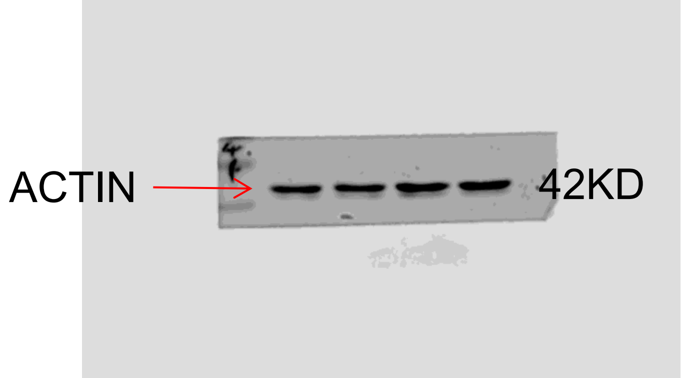
ACTIN
42KD

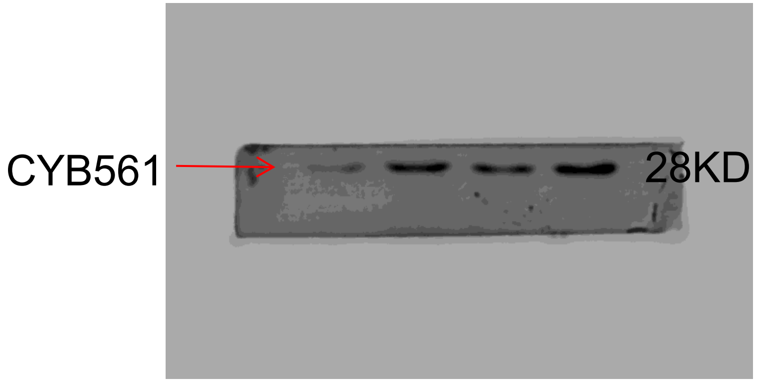
CYB561
28KD

Fig.5A

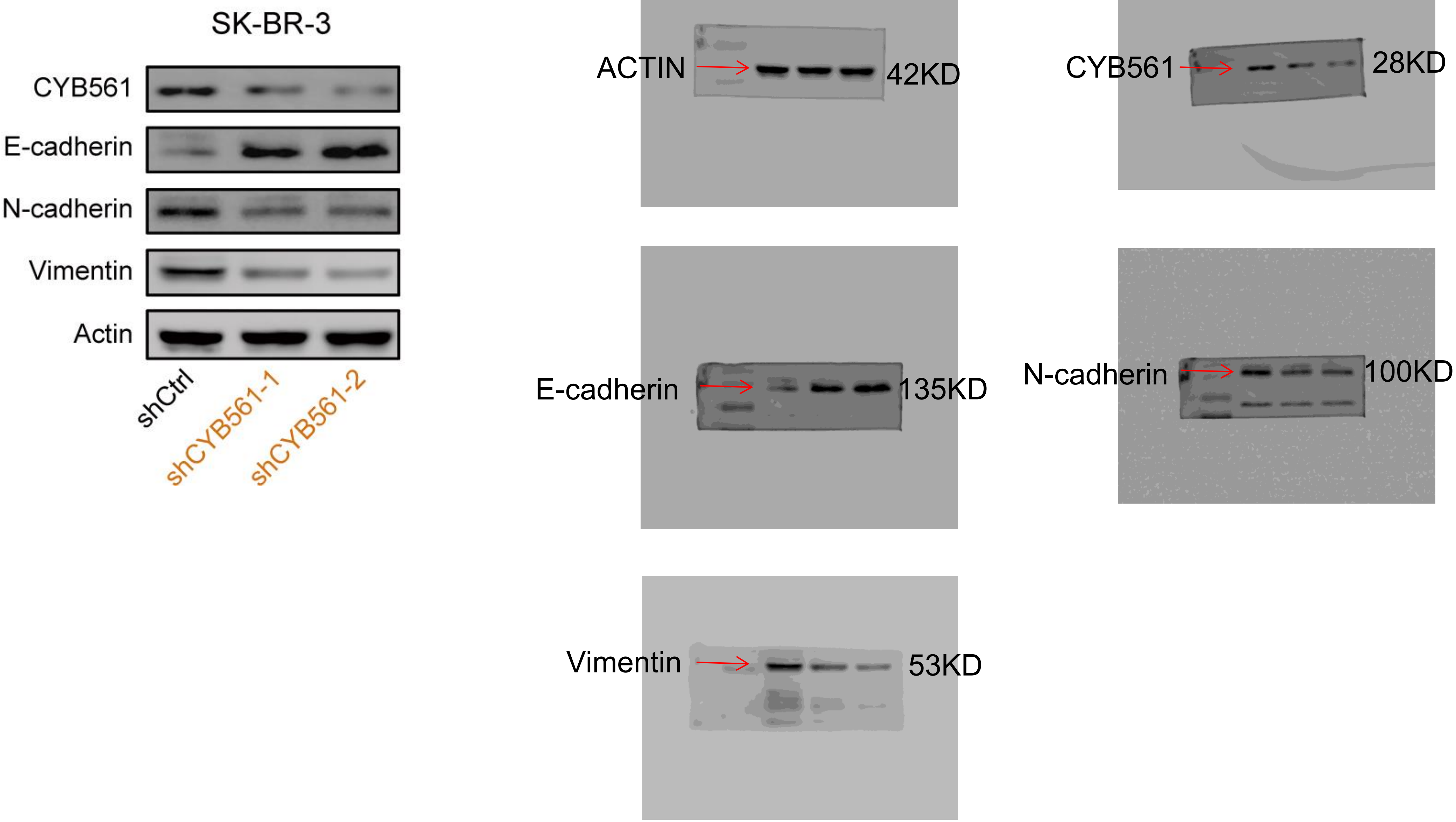
SK-BR-3
CYB561
E-cadherin
N-cadherin
Vimentin
Actin
shCtrl
shCYB561-1
shCYB561-2
ACTIN
42KD
CYB561
28KD
E-cadherin
135KD
N-cadherin
100KD
Vimentin
53KD

Fig.5B

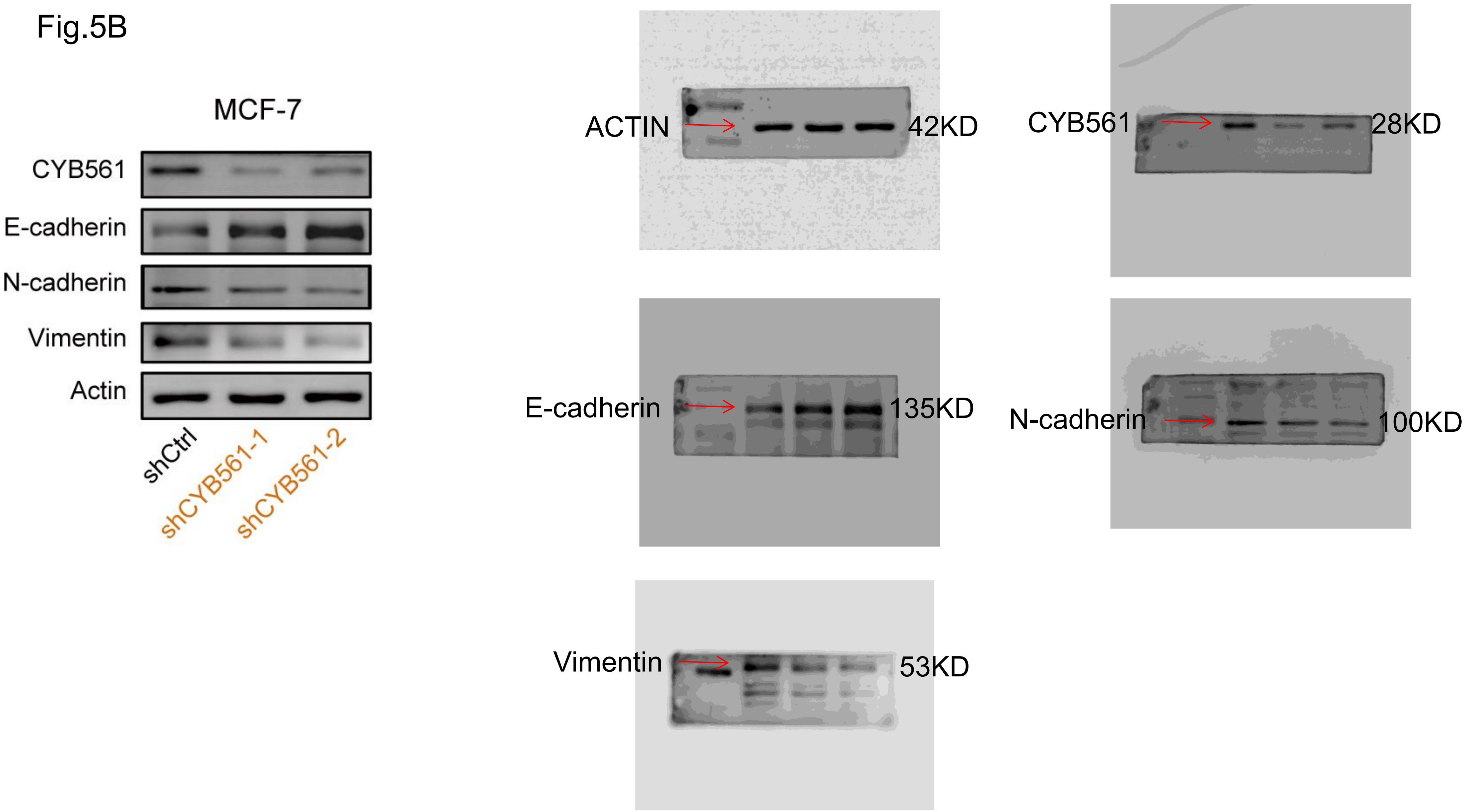
MCF-7
CYB561
E-cadherin
N-cadherin
Vimentin
Actin
shCtrl
shCYB561-1
shCYB561-2
ACTIN
42KD
CYB561
28KD
E-cadherin
135KD
N-cadherin
100KD
Vimentin
53KD

Fig.5C

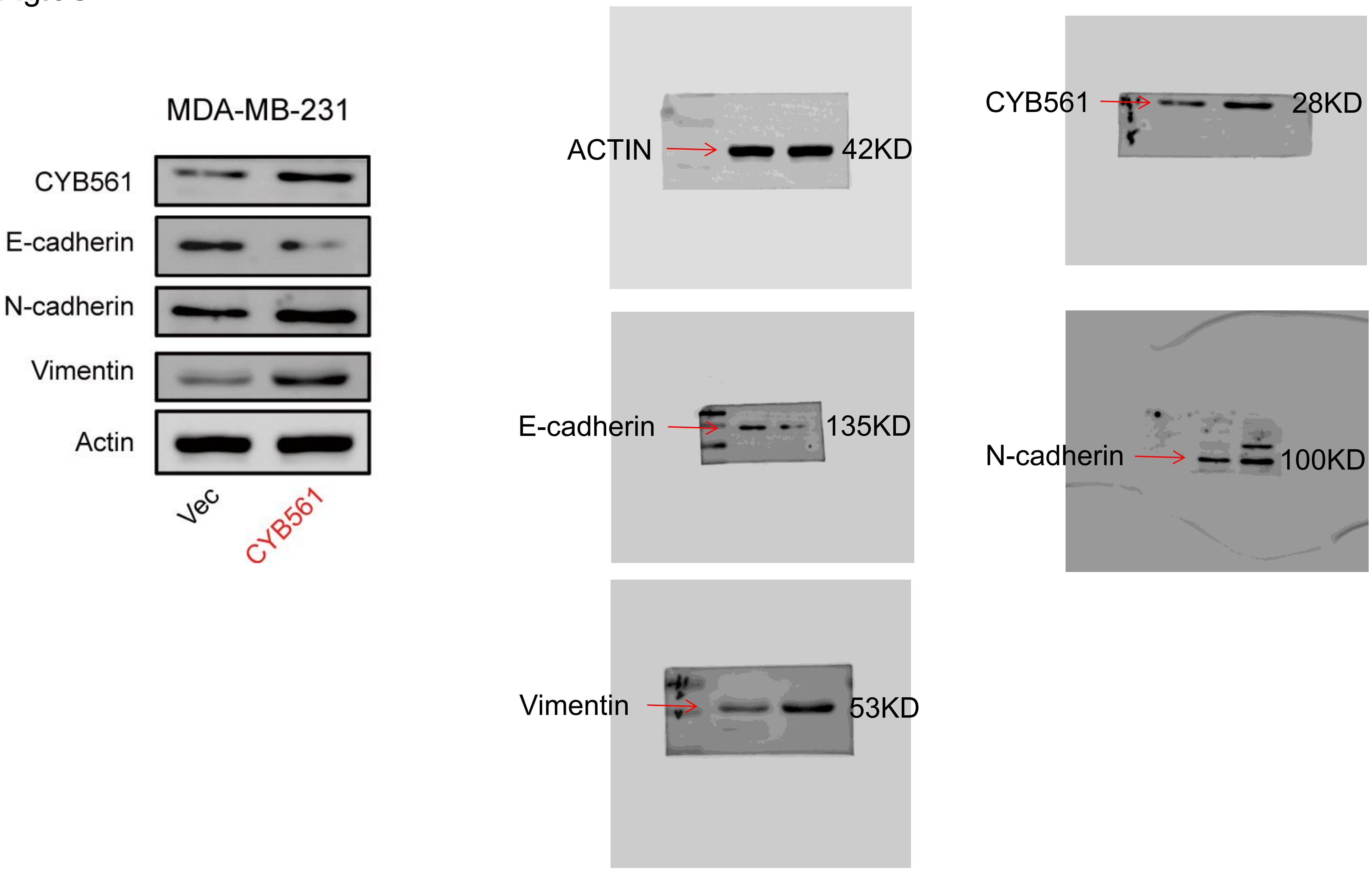
MDA-MB-231
CYB561
E-cadherin
N-cadherin
Vimentin
Actin
Vec
CYB561
ACTIN
42KD
CYB561
28KD
E-cadherin
135KD
N-cadherin
100KD
Vimentin
53KD

Fig.5D

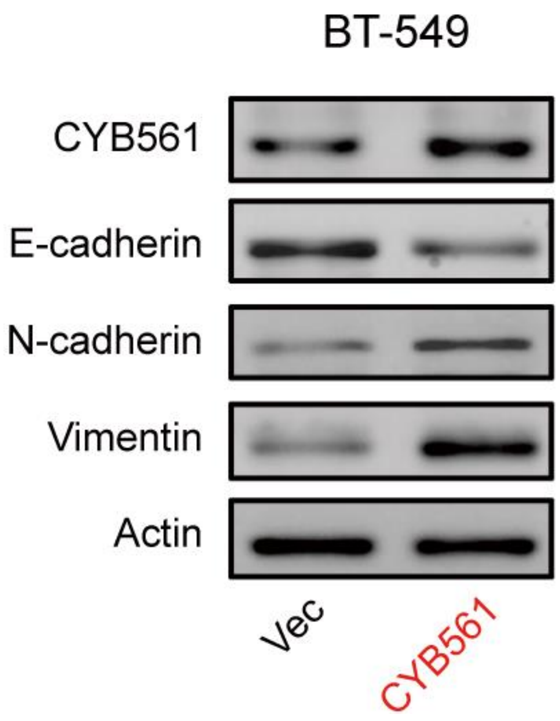
BT-549
CYB561
E-cadherin
N-cadherin
Vimentin
Actin
Vec
CYB561

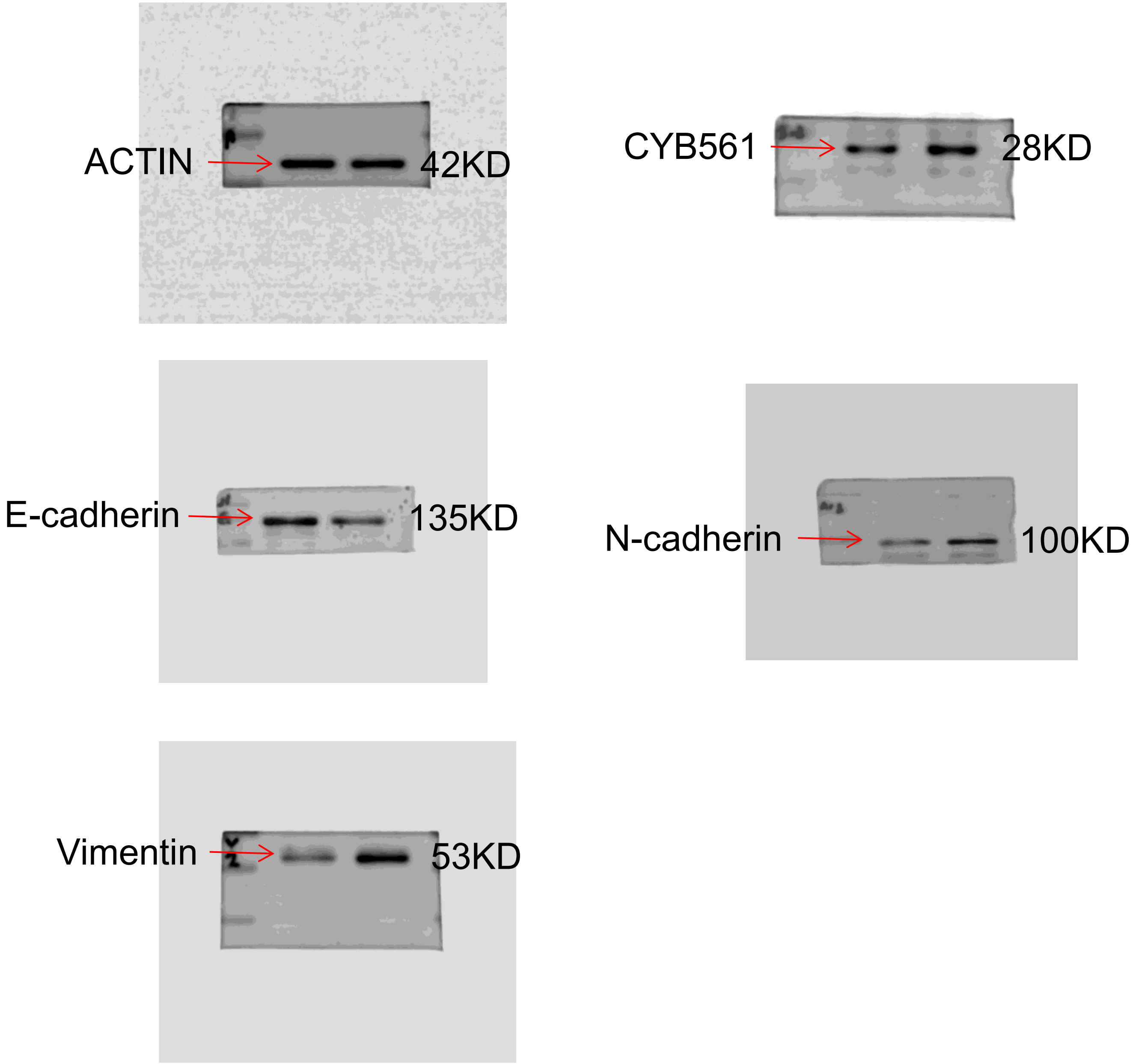
ACTIN
42KD
CYB561
28KD
E-cadherin
135KD
N-cadherin
100KD
Vimentin
53KD

Fig.7A

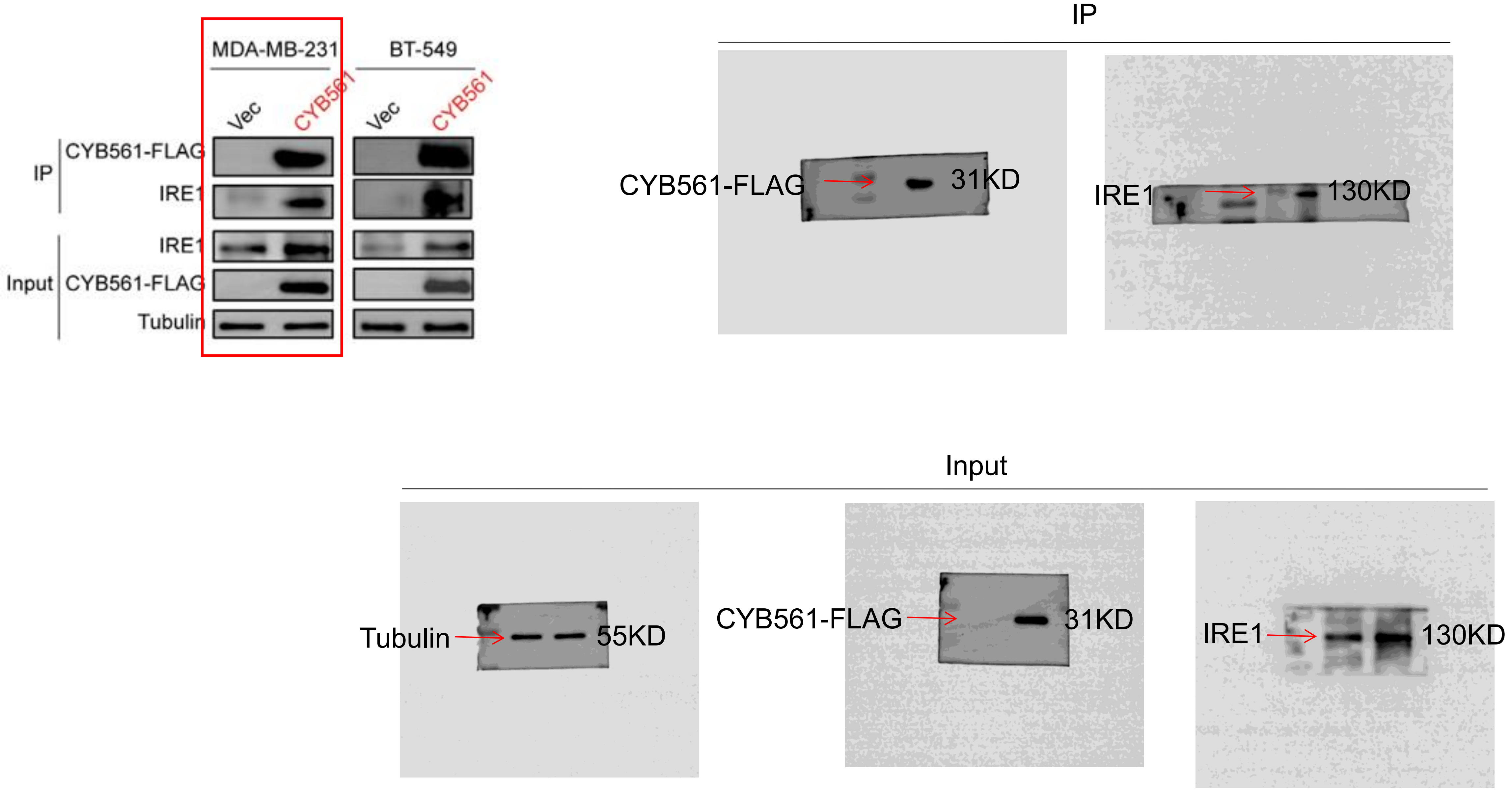

MDA-MB-231
BT-549
Vec
CYB561
Vec
CYB561
IP
CYB561-FLAG
IRE1
IRE1
Input
CYB561-FLAG
Tubulin
IP
CYB561-FLAG
31KD
IRE1
130KD
Input
Tubulin
55KD
CYB561-FLAG
31KD
IRE1
130KD

Fig.7A

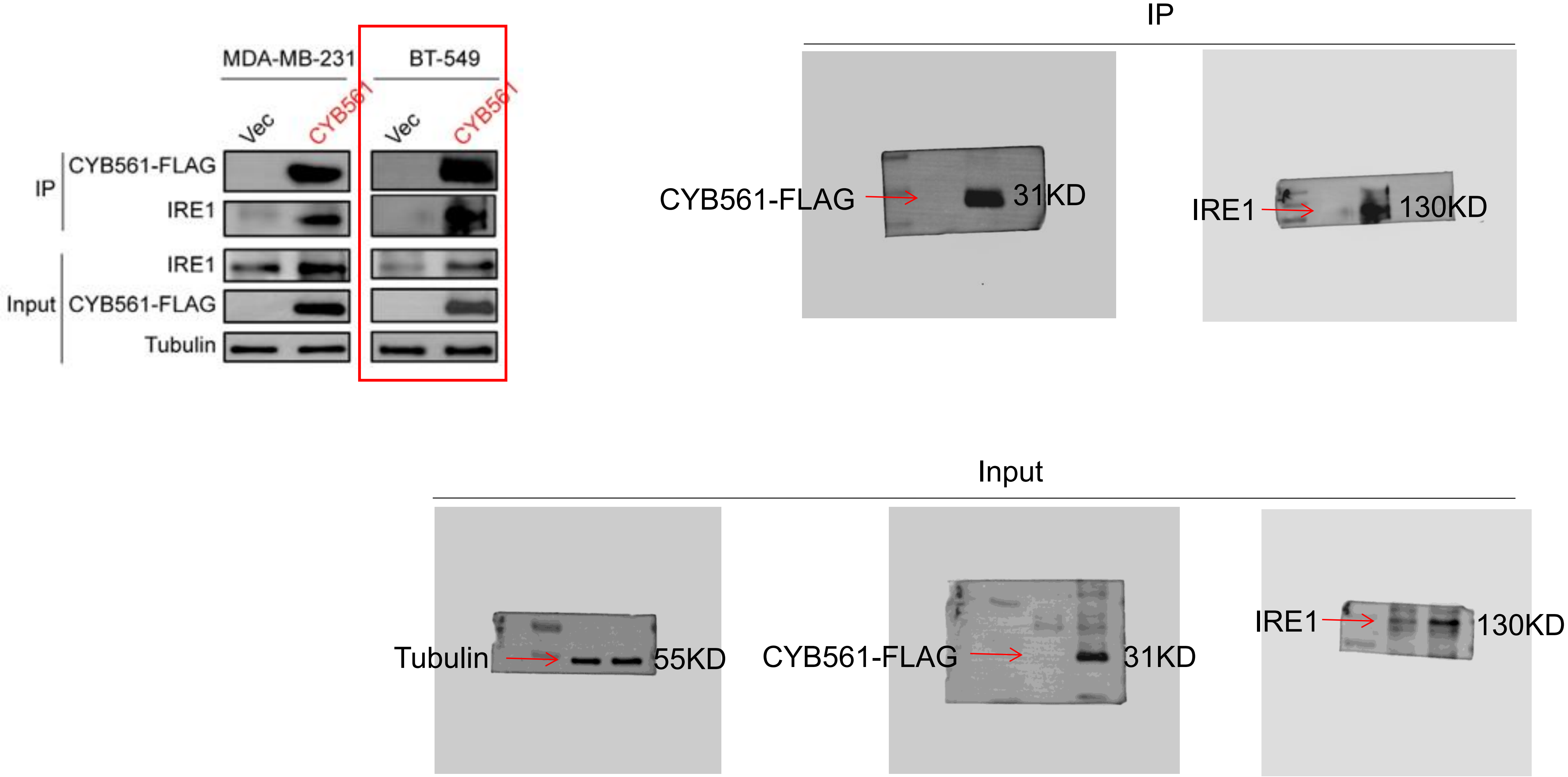
MDA-MB-231
BT-549
Vec
CYB561
Vec
CYB561
IP
CYB561-FLAG
IRE1
Input
IRE1
CYB561-FLAG
Tubulin
IP
CYB561-FLAG
31KD
IRE1
130KD
Input
Tubulin
55KD
CYB561-FLAG
31KD
IRE1
130KD

Fig.7D

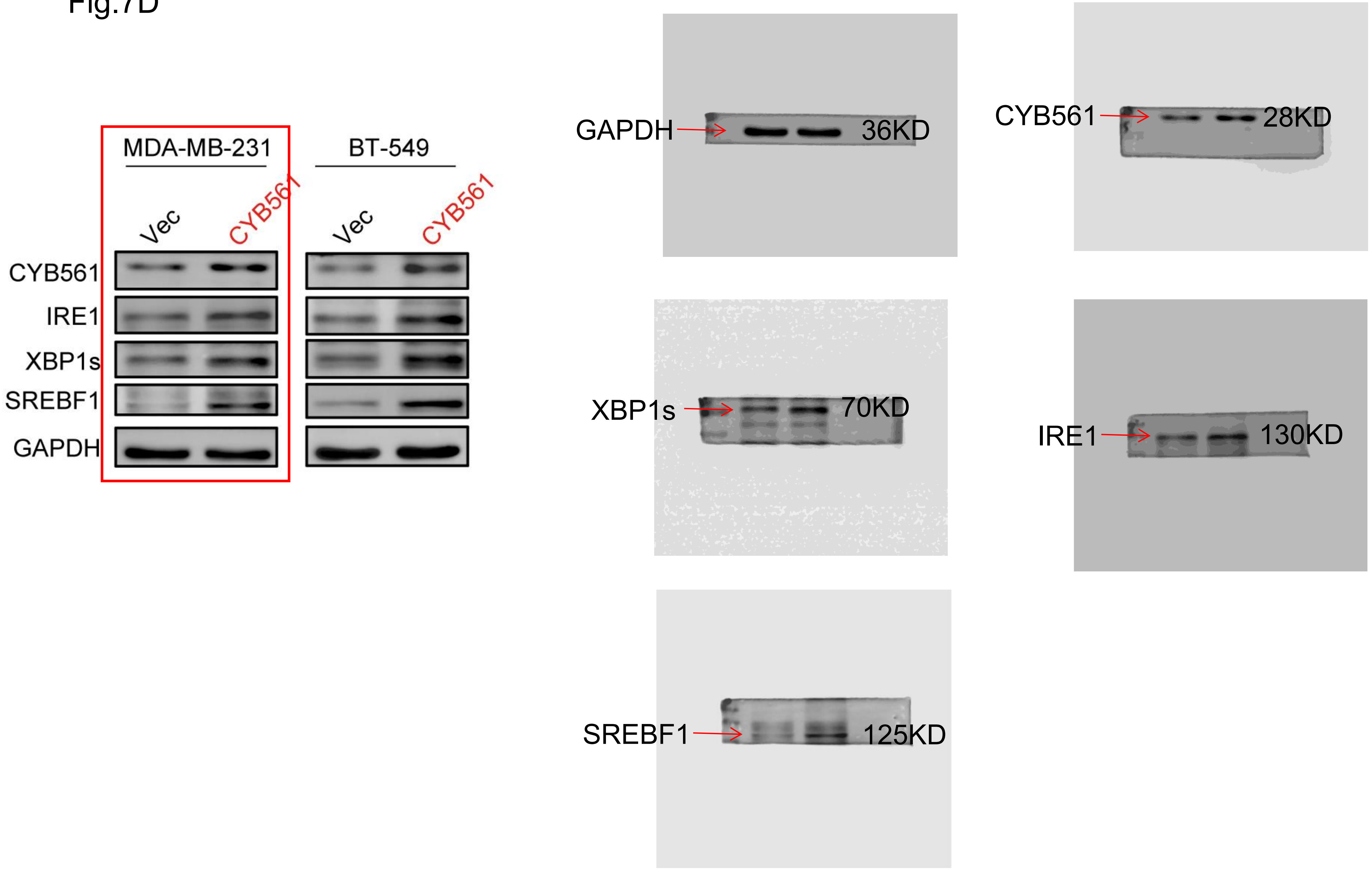

MDA-MB-231
BT-549
Vec
CYB561
Vec
CYB561
CYB561
IRE1
XBP1s
SREBF1
GAPDH
GAPDH
36KD
CYB561
28KD
XBP1s
70KD
IRE1
130KD
SREBF1
125KD

Fig.7D

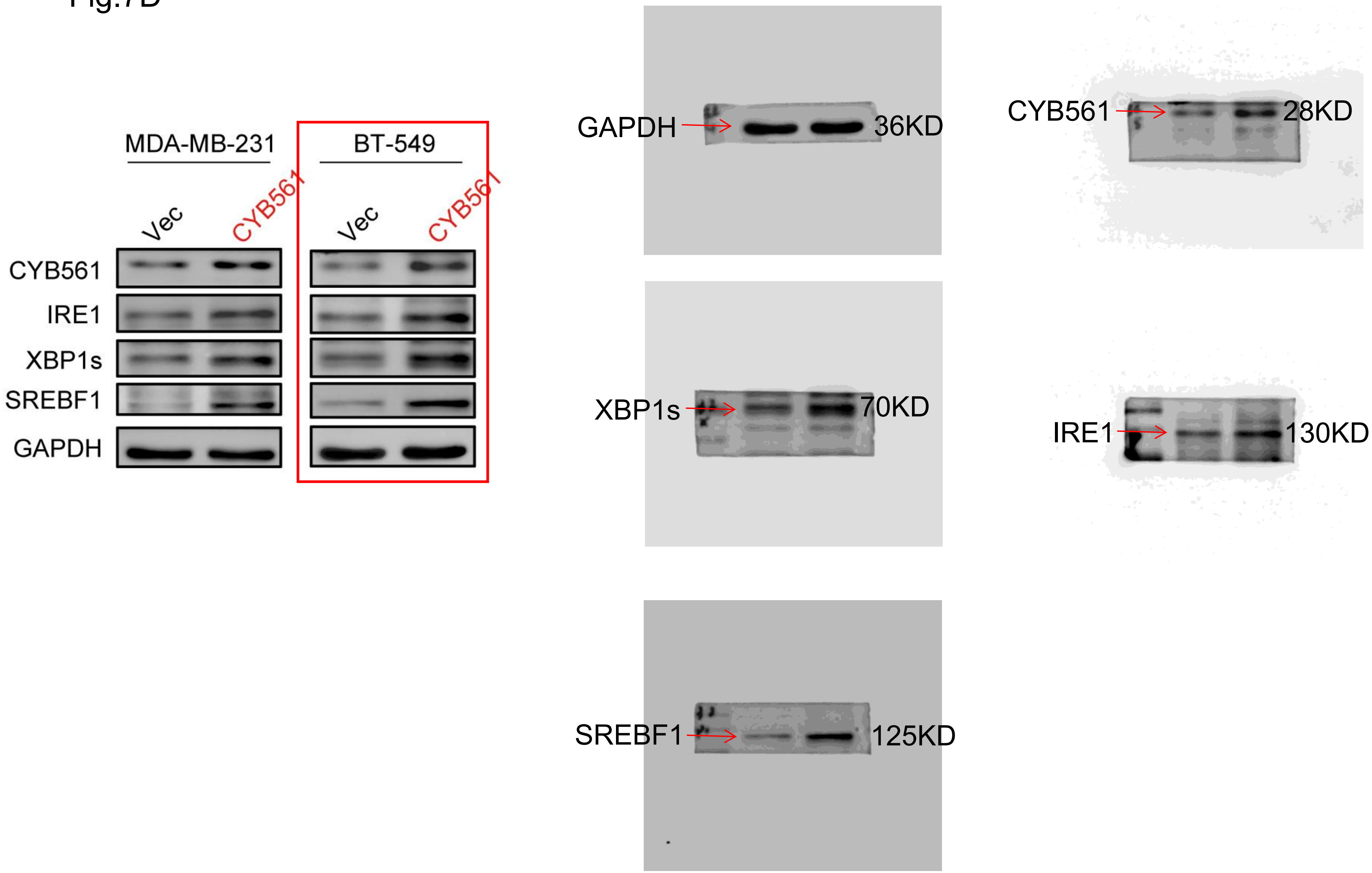
MDA-MB-231
BT-549
Vec
CYB561
Vec
CYB561
CYB561
IRE1
XBP1s
SREBF1
GAPDH
GAPDH
36KD
CYB561
28KD
XBP1s
70KD
IRE1
130KD
SREBF1
125KD

Fig.7E

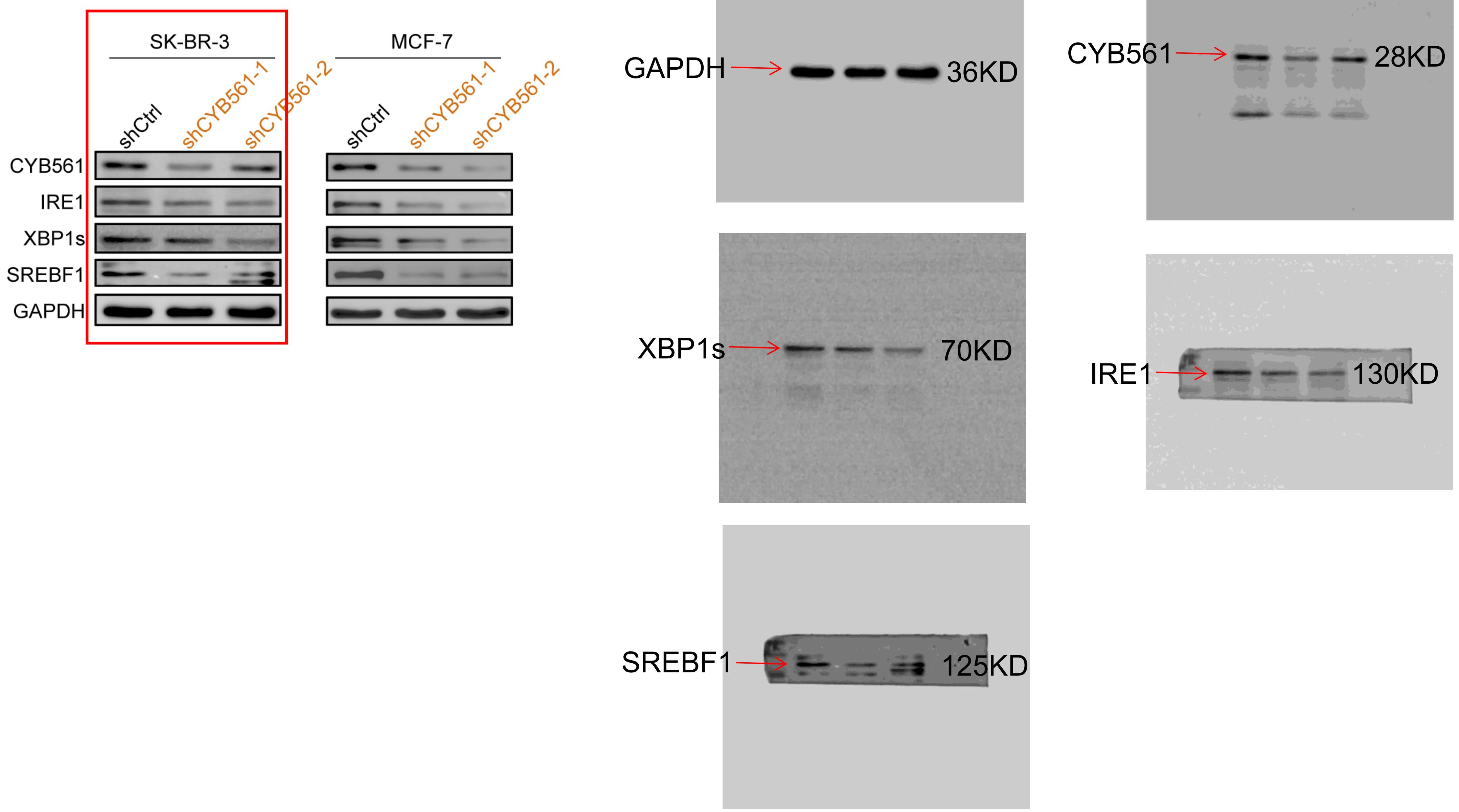
SK-BR-3
MCF-7
shCtrl
shCYB561-1
shCYB561-2
CYB561
IRE1
XBP1s
SREBF1
GAPDH
GAPDH
36KD
CYB561
28KD
XBP1s
70KD
IRE1
130KD
SREBF1
125KD

Fig.7E

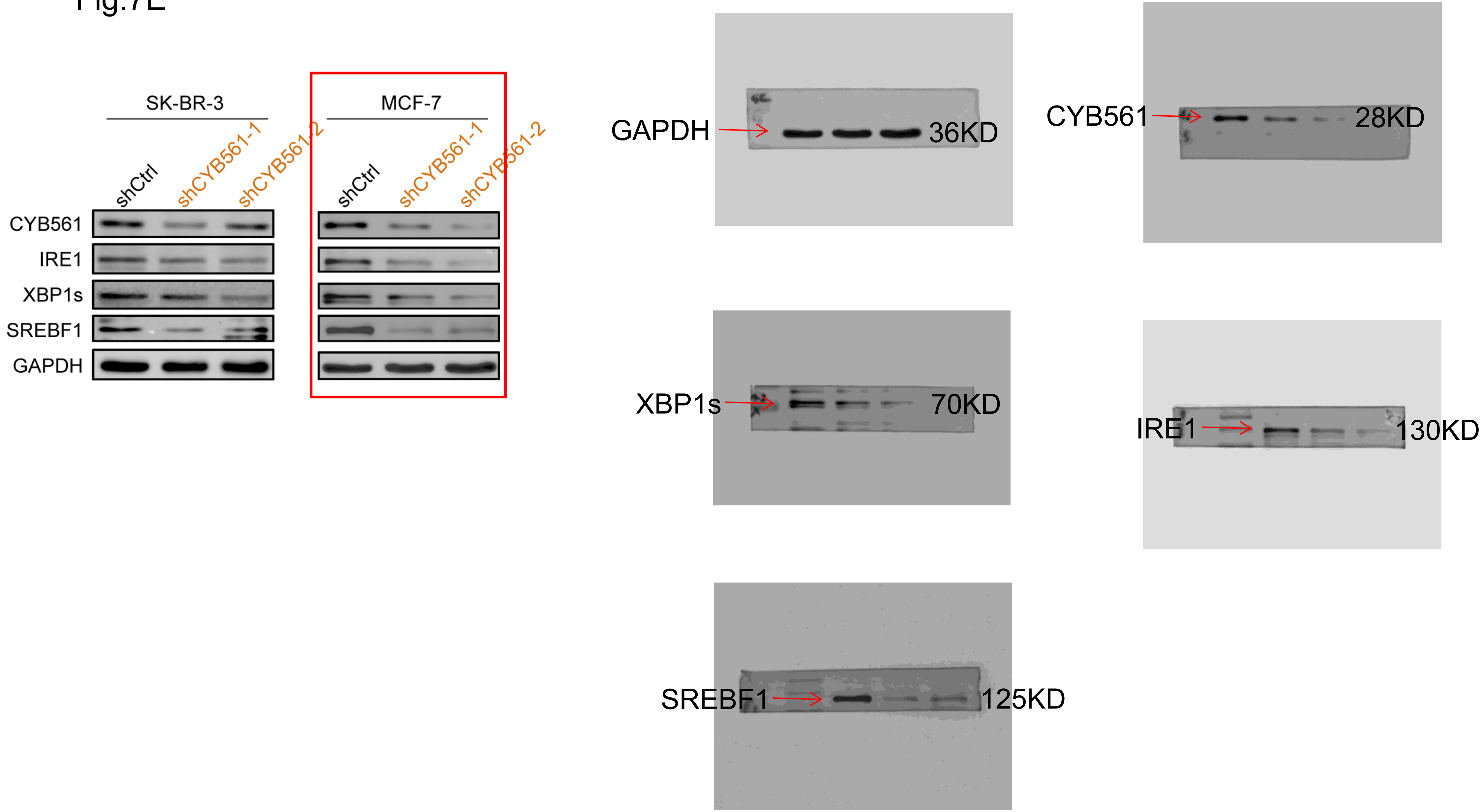
SK-BR-3
MCF-7
shCtrl
shCYB561-1
shCYB561-2
shCtrl
shCYB561-1
shCYB561-2
CYB561
IRE1
XBP1s
SREBF1
GAPDH
GAPDH
36KD
CYB561
28KD
XBP1s
70KD
IRE1
130KD
SREBF1
125KD

Fig.7F

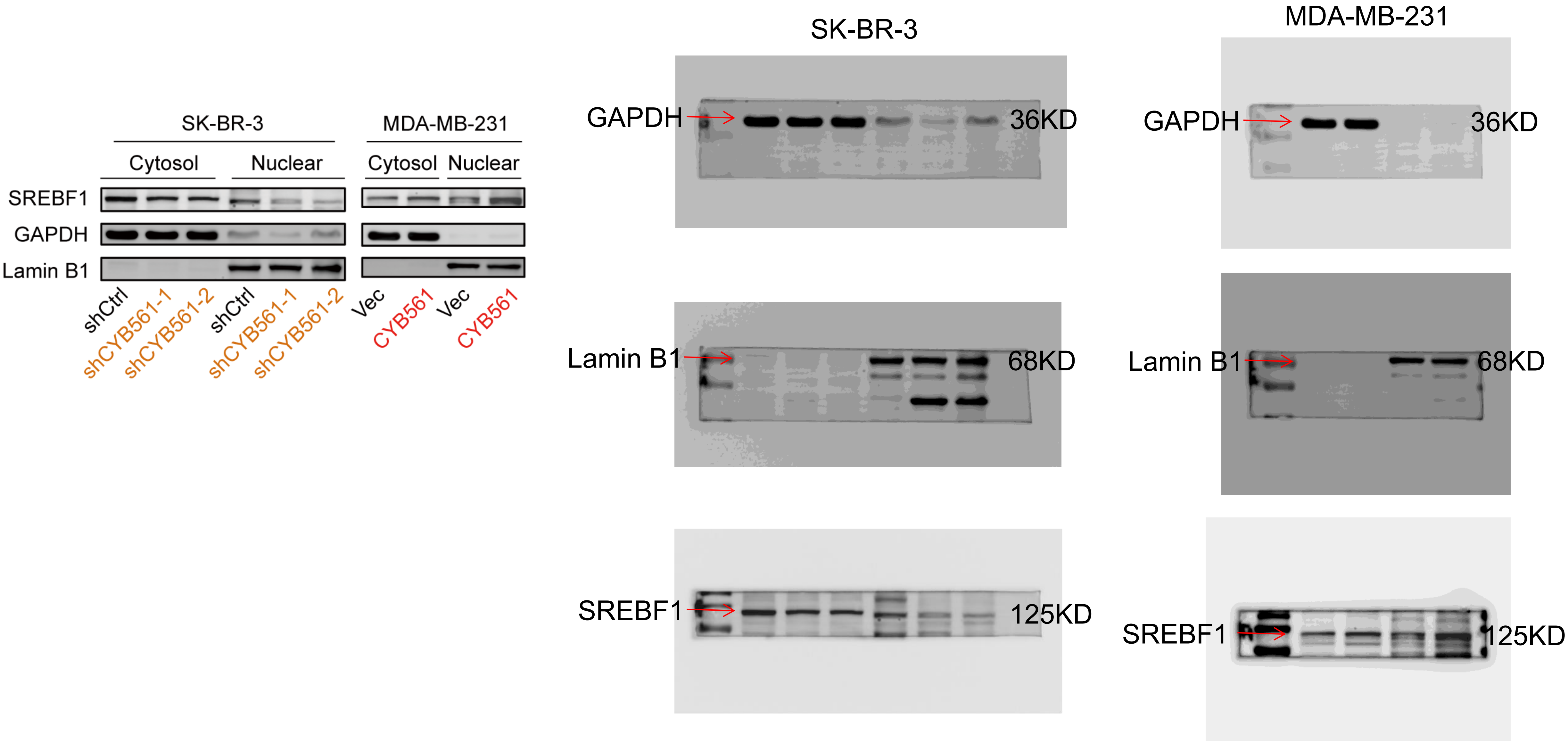

SK-BR-3
MDA-MB-231
Cytosol
Nuclear
Cytosol
Nuclear
SREBF1
GAPDH
Lamin B1
shCtrl
shCYB561-1
shCYB561-2
shCtrl
shCYB561-1
shCYB561-2
Vec
CYB561
Vec
CYB561
SK-BR-3
MDA-MB-231
GAPDH
36KD
GAPDH
36KD
Lamin B1
68KD
Lamin B1
68KD
SREBF1
125KD
SREBF1
125KD

Fig.8A

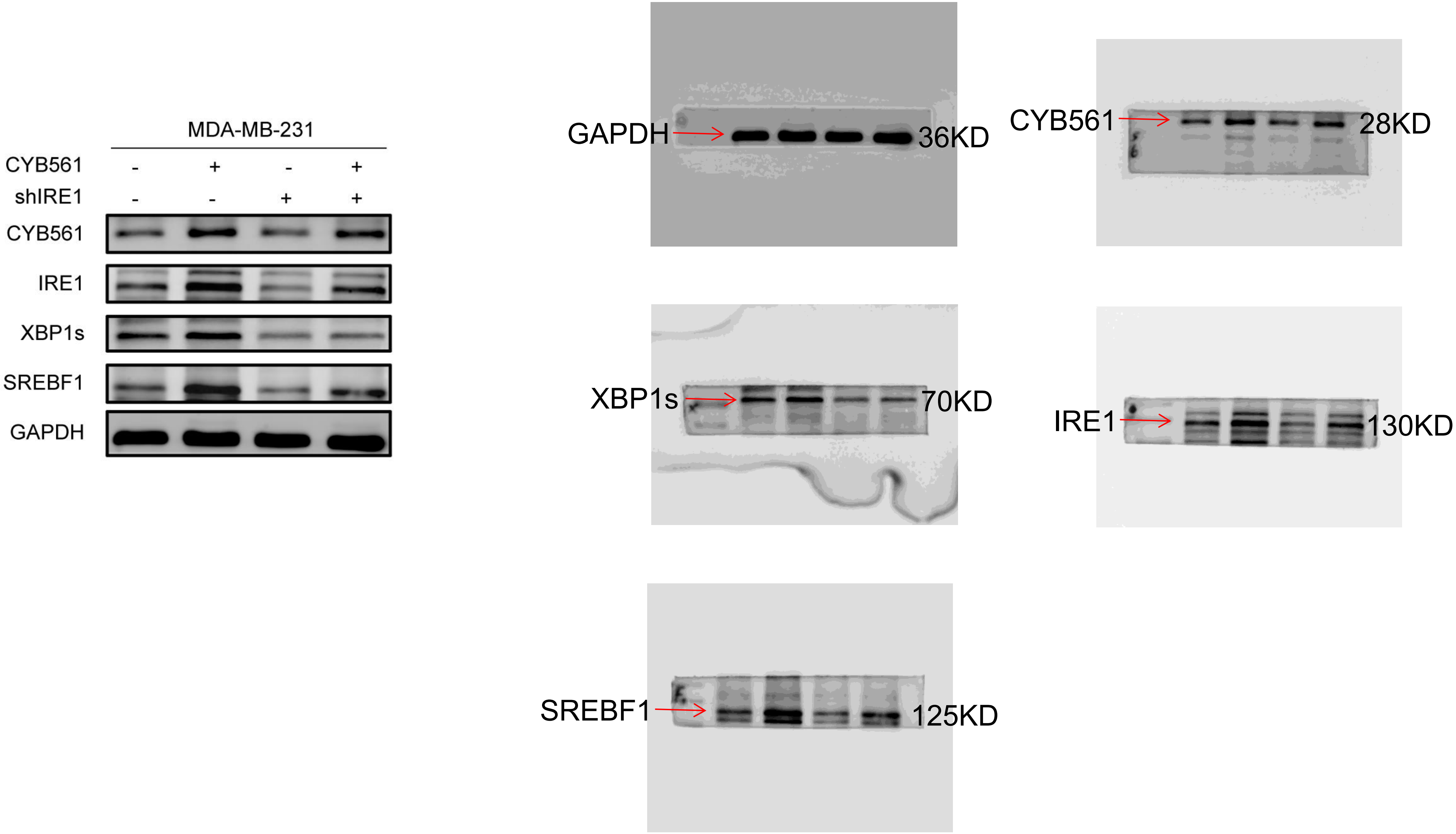
MDA-MB-231
CYB561
shIRE1
CYB561
IRE1
XBP1s
SREBF1
GAPDH
GAPDH
36KD
CYB561
28KD
XBP1s
70KD
IRE1
130KD
SREBF1
125KD

Fig.8F

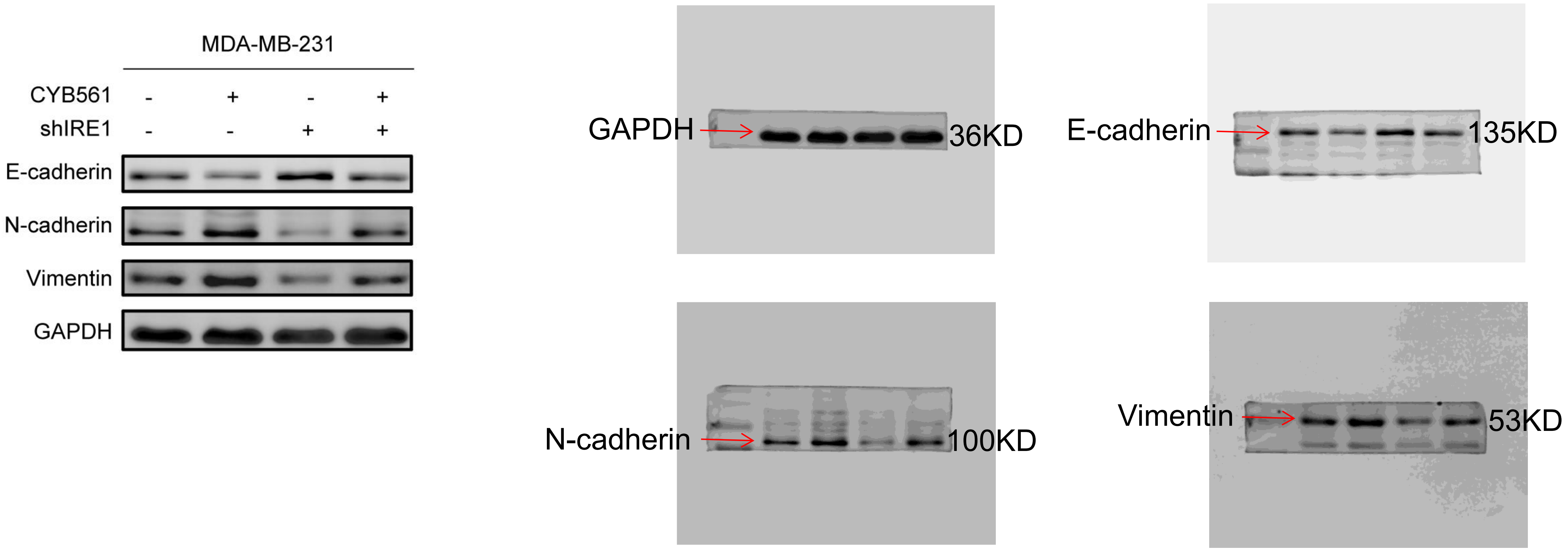
MDA-MB-231
CYB561 - + - +
shIRE1 - - + +
E-cadherin
N-cadherin
Vimentin
GAPDH
GAPDH
36KD
E-cadherin
135KD
N-cadherin
100KD
Vimentin
53KD

Fig.9C

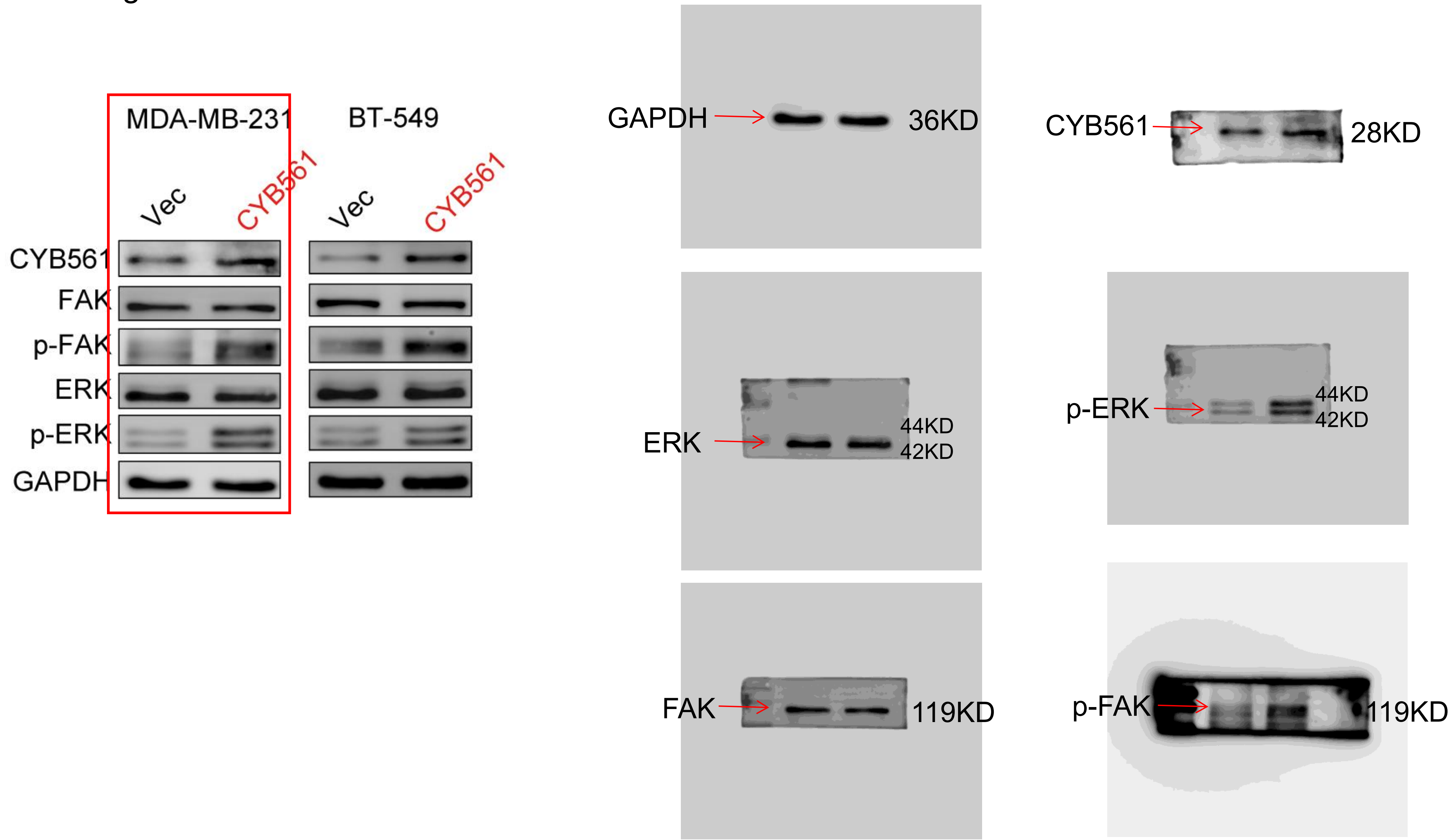
MDA-MB-231
BT-549
Vec
CYB561
Vec
CYB561
CYB561
FAK
p-FAK
ERK
p-ERK
GAPDH
GAPDH
36KD
CYB561
28KD
ERK
44KD
42KD
p-ERK
44KD
42KD
FAK
119KD
p-FAK
119KD

Fig.9C

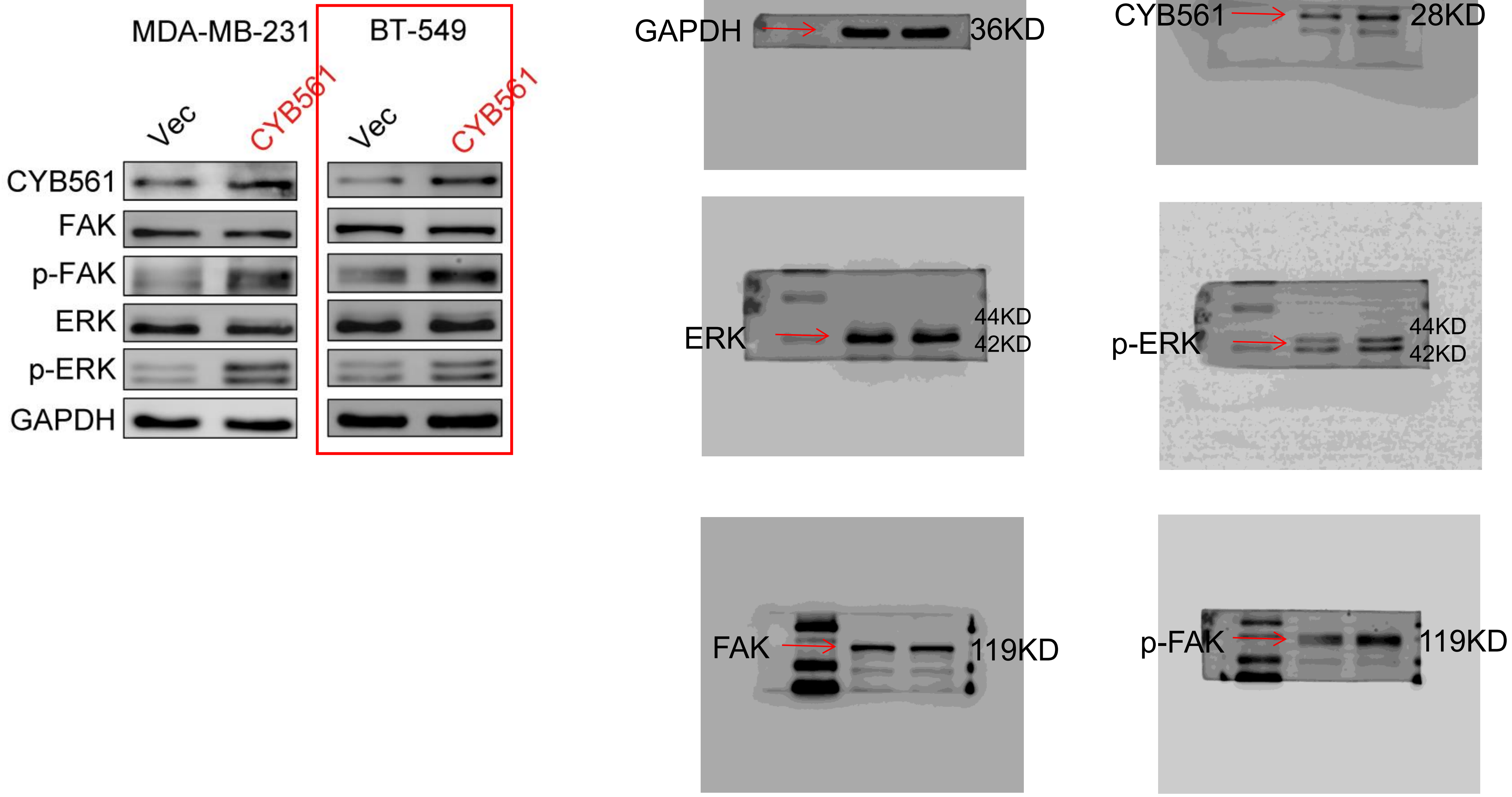
MDA-MB-231
BT-549
Vec
CYB561
Vec
CYB561
CYB561
FAK
p-FAK
ERK
p-ERK
GAPDH
GAPDH
36KD
CYB561
28KD
ERK
44KD
42KD
p-ERK
44KD
42KD
FAK
119KD
p-FAK
119KD

Fig.9D

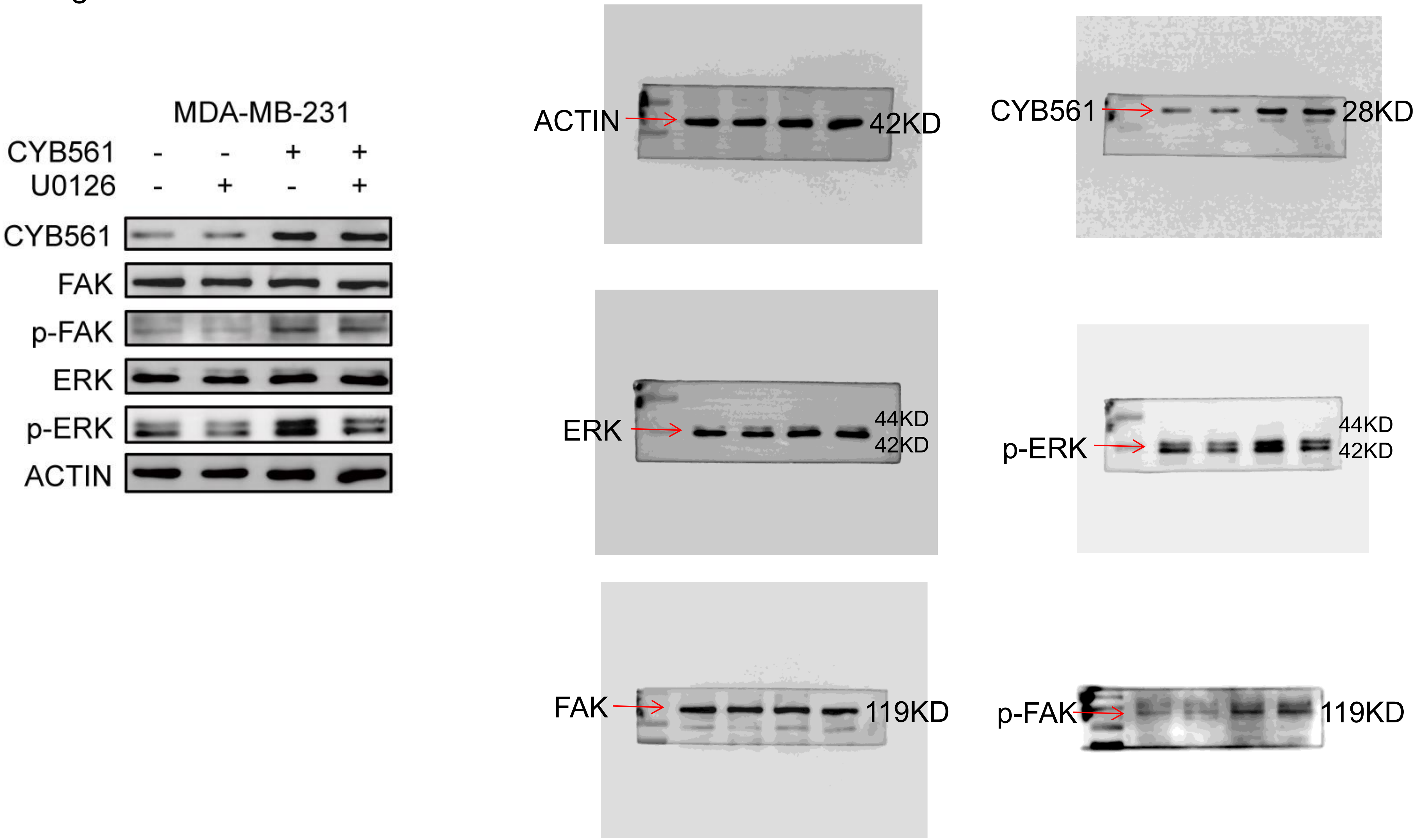
MDA-MB-231
CYB561 - - + +
U0126 - + - +
CYB561
FAK
p-FAK
ERK
p-ERK
ACTIN
ACTIN
42KD
CYB561
28KD
ERK
44KD
42KD
p-ERK
44KD
42KD
FAK
119KD
p-FAK
119KD

Fig.9I

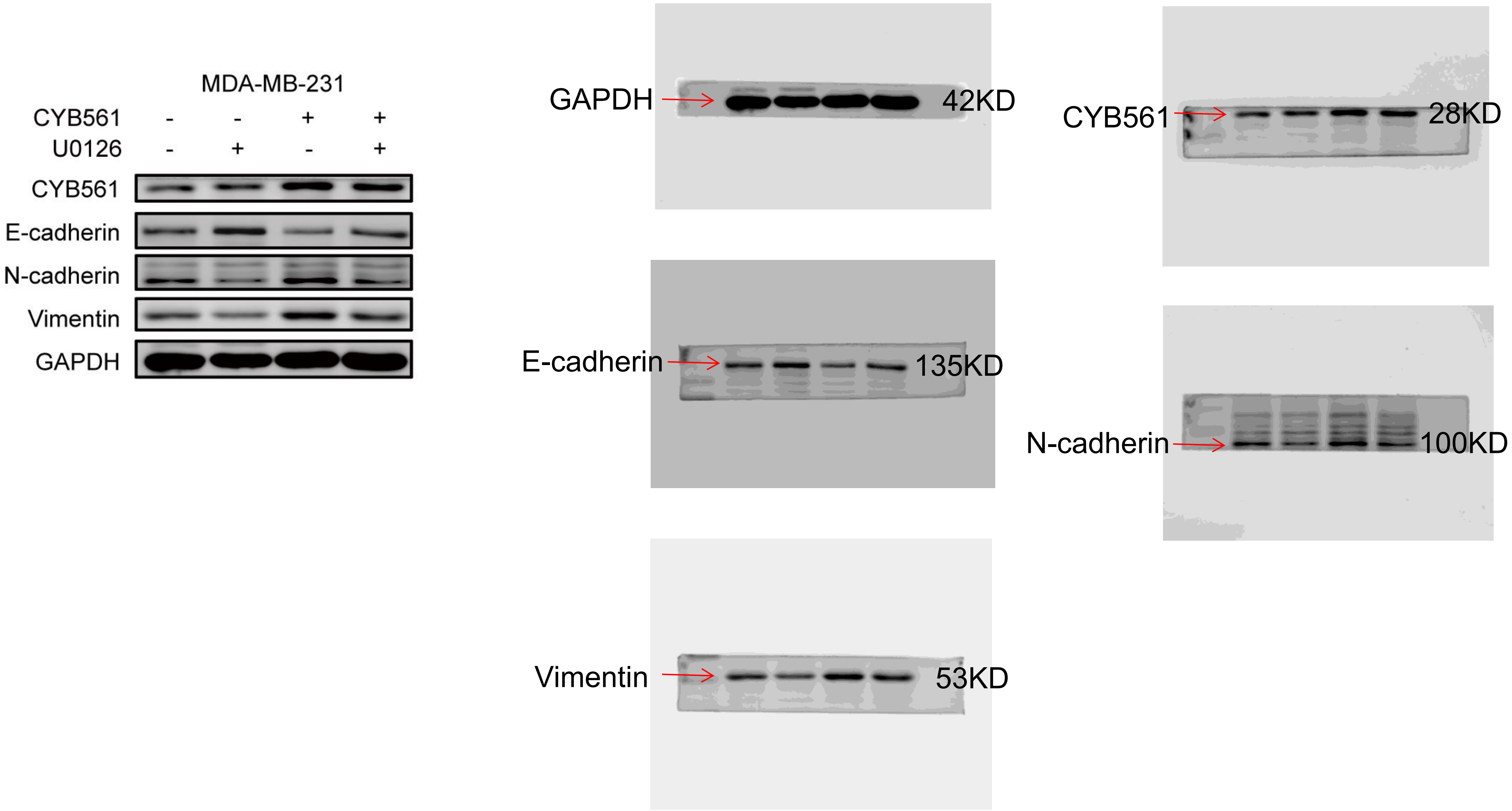
MDA-MB-231
CYB561 - - + +
U0126 - + - +
CYB561
E-cadherin
N-cadherin
Vimentin
GAPDH
GAPDH 42KD
CYB561 28KD
E-cadherin 135KD
N-cadherin 100KD
Vimentin 53KD

Supplementary Fig.3B

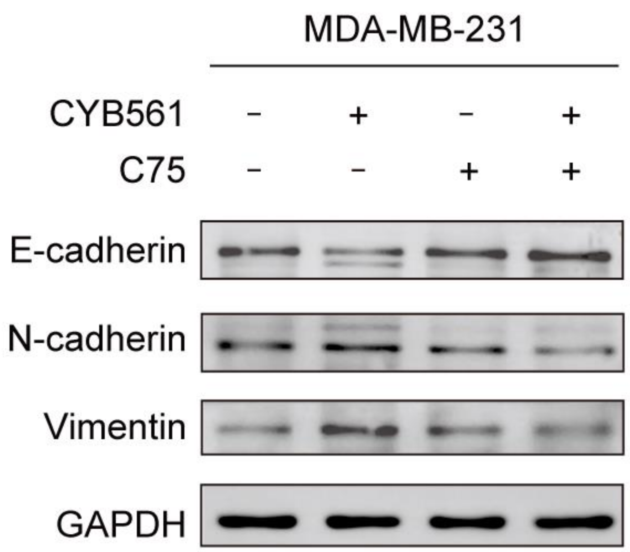

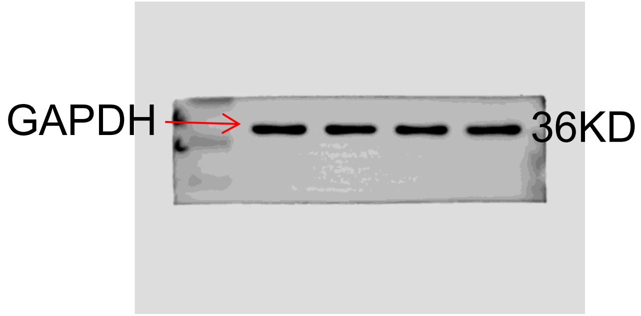

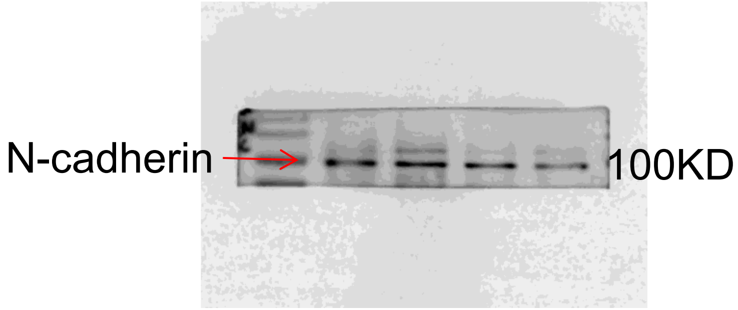

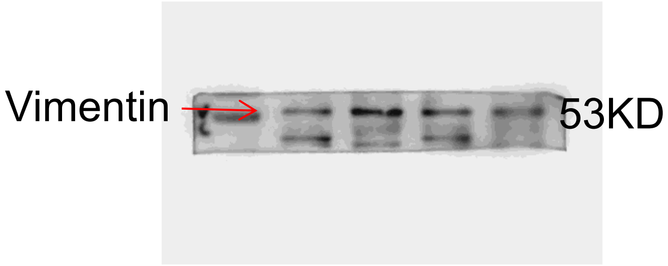

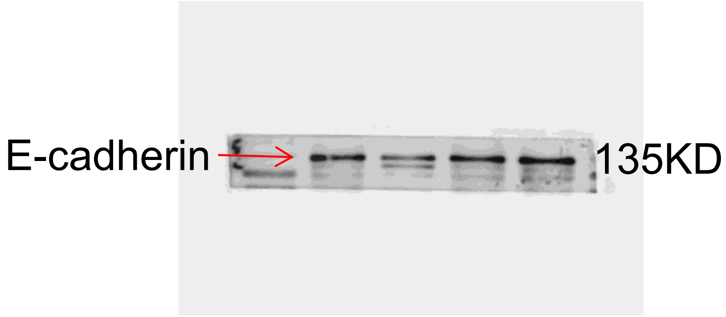

Supplementary Fig.3C

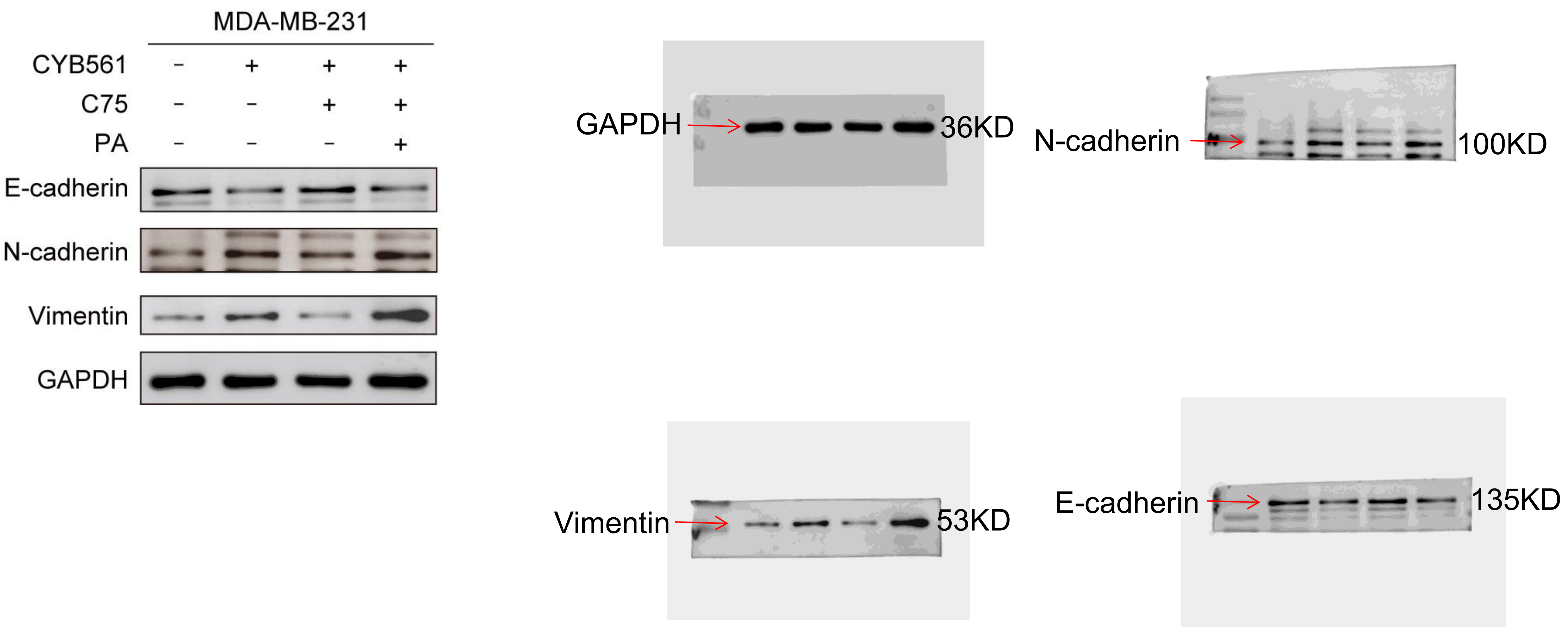

Supplementary Fig.4B

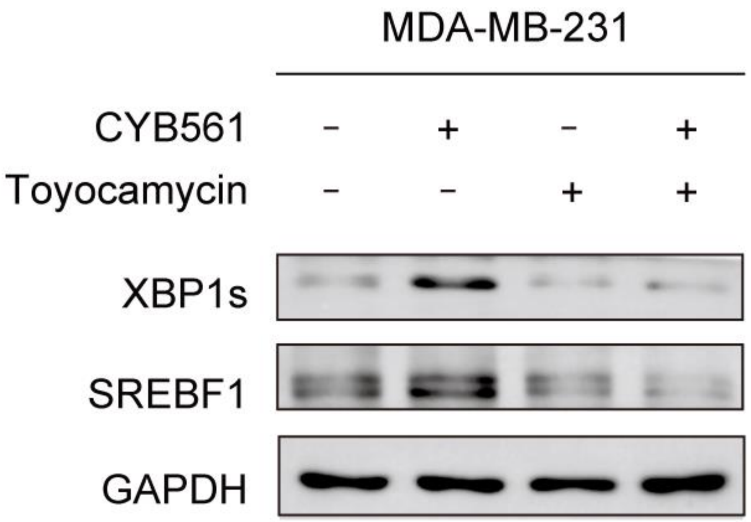

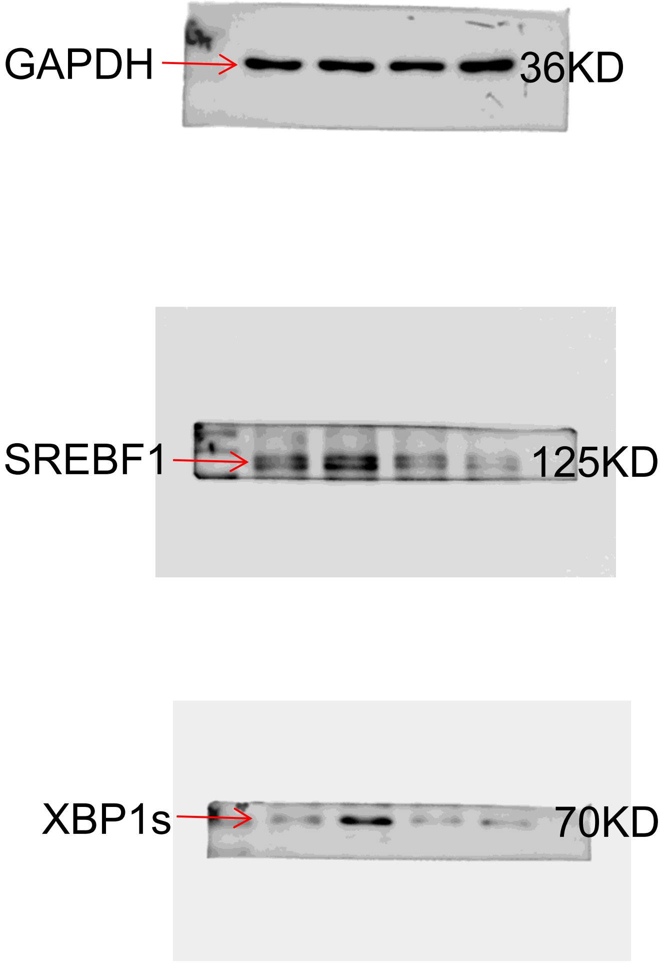

Supplementary Fig.5

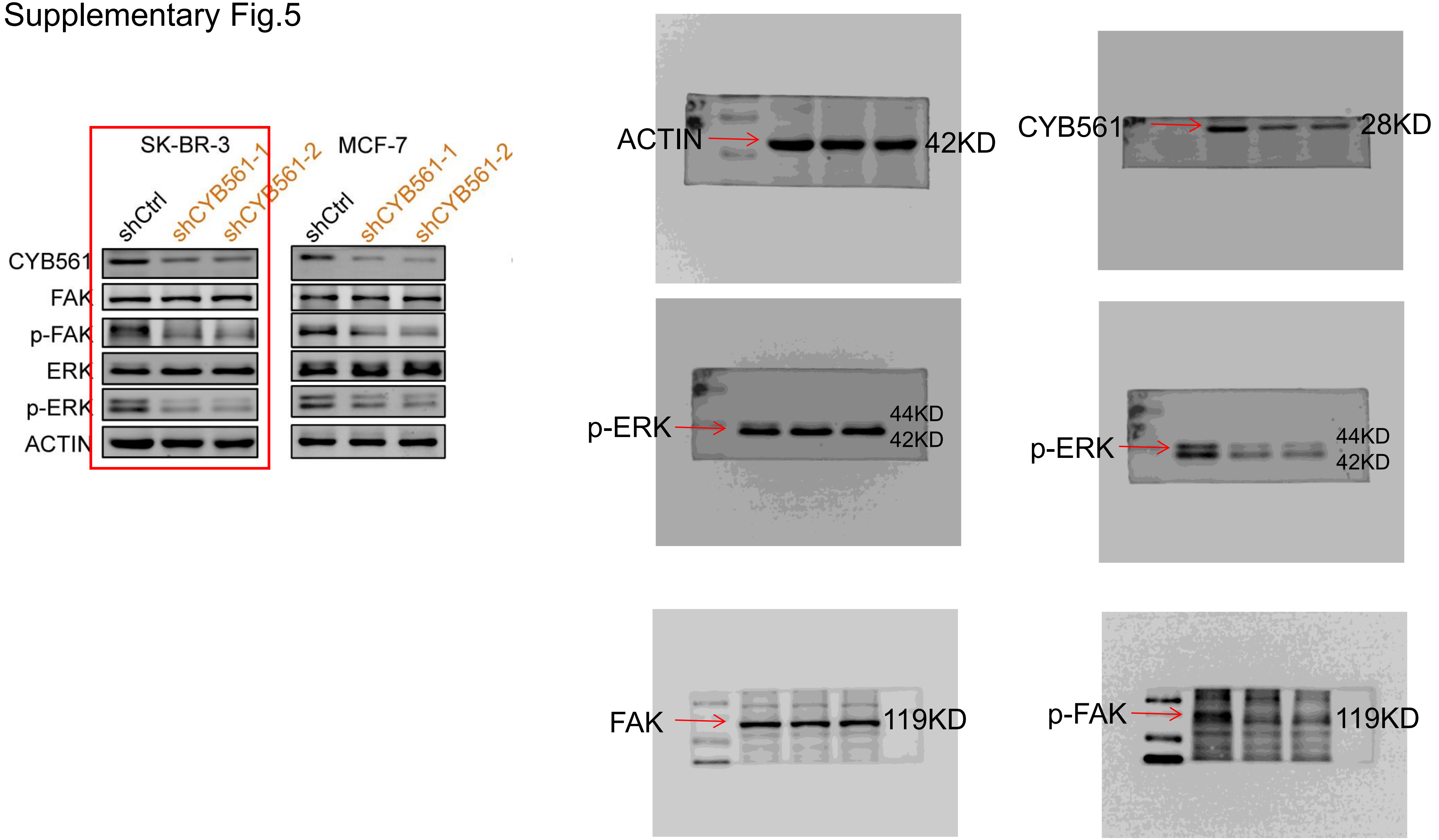

Supplementary Fig.5

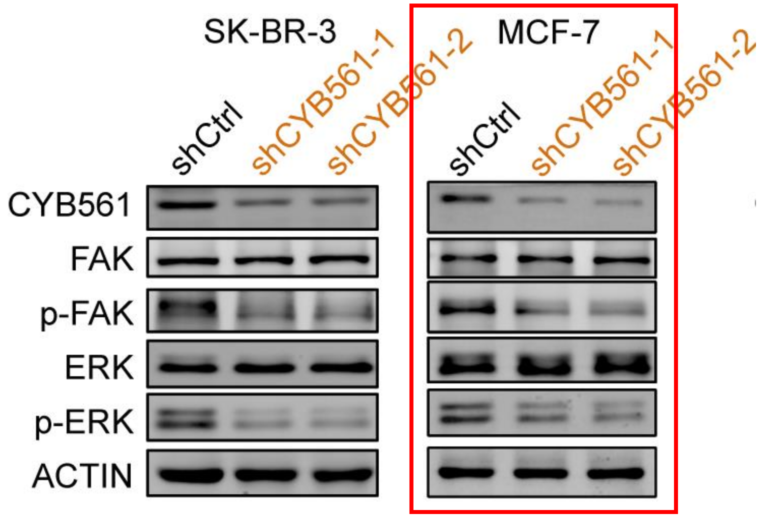

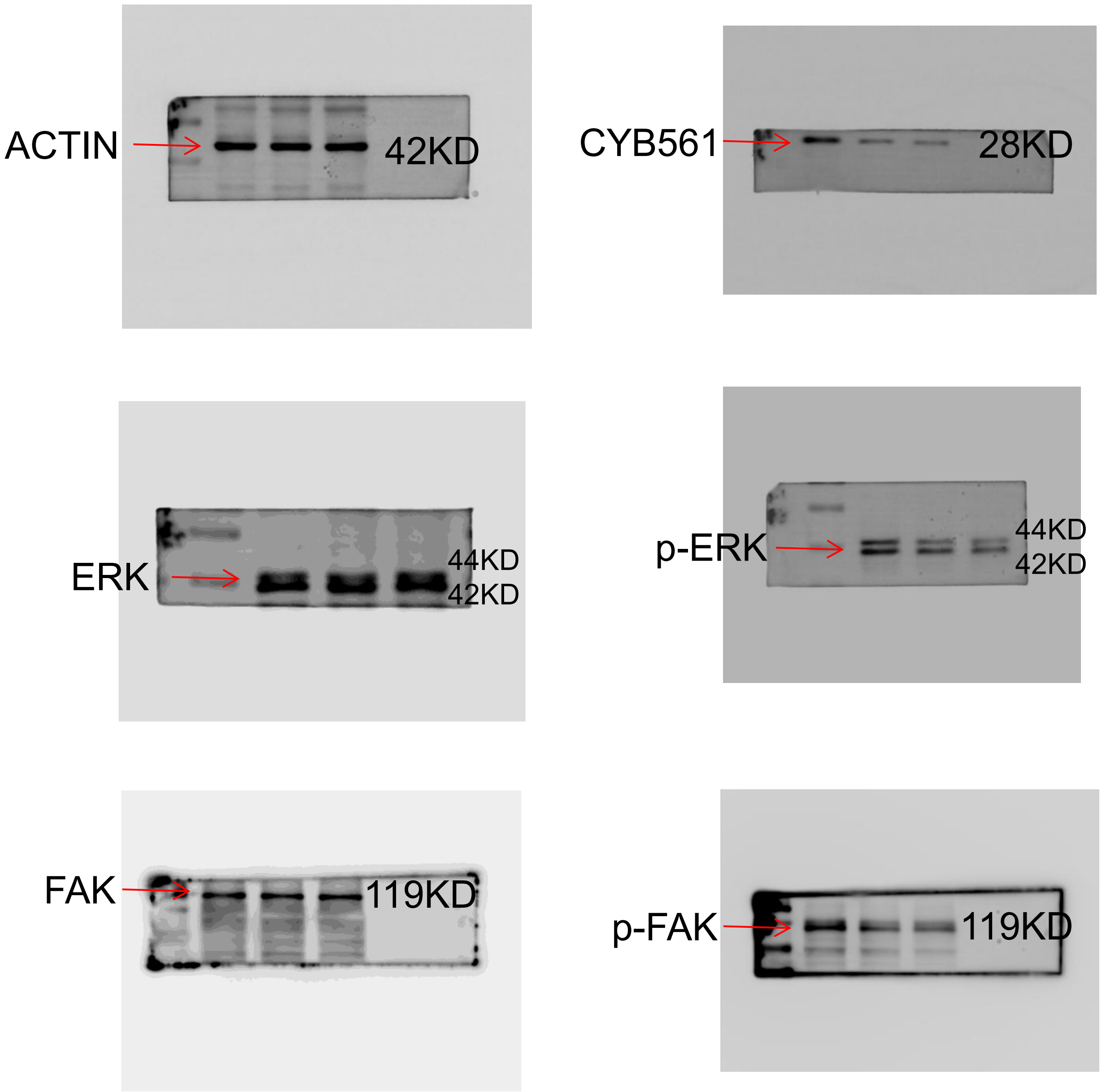

Supplement: Supplementary file 2 — Original western blot images [file 41420_2026_3101_MOESM2_ESM.pdf]
